# Serum extracellular vesicle microRNAs as potential biomarkers to predict pembrolizumab response and prognosis in metastatic non-small cell lung cancer patients

**DOI:** 10.3389/fimmu.2025.1540906

**Published:** 2025-06-04

**Authors:** Daniela Lamorte, Luciana De Luca, Alfredo Tartarone, Stefania Trino, Irene Giulivo, Angelo De Stradis, Maddalena Maietti, Antonella Caivano, Ilaria Laurenzana

**Affiliations:** ^1^ Laboratory of Preclinical and Translational Research, Istituto di Ricovero e Cura a Carattere Scientifico (IRCCS) Centro di Riferimento Oncologico della Basilicata (CROB), Rionero in Vulture, Italy; ^2^ Unit of Clinical Pathology, Istituto di Ricovero e Cura a Carattere Scientifico (IRCCS) Centro di Riferimento Oncologico della Basilicata (CROB), Rionero in Vulture, Italy; ^3^ Department of Onco-Hematology, Division of Medical Oncology, Istituto di Ricovero e Cura a Carattere Scientifico (IRCCS) Centro di Riferimento Oncologico della Basilicata (CROB), Rionero in Vulture, Italy; ^4^ Trial Office, Istituto di Ricovero e Cura a Carattere Scientifico (IRCCS) Centro di Riferimento Oncologico della Basilicata (CROB), Rionero in Vulture, Italy; ^5^ Institute for Sustainable Plant Protection, National Research Council (CNR), Bari, Italy

**Keywords:** NSCLC, immunotherapy, liquid biopsy, biomarkers, extracellular vesicles, microRNAs, response prediction, prognosis

## Abstract

**Introduction:**

Circulating Extracellular Vesicles (cEVs) could represent new non-invasive biomarkers for diagnosis and prognosis in tumors. In the context of Non-Small Cell Lung Cancer (NSCLC) immunotherapy there’s a great need for novel predictive and prognostic biomarkers. This study aims to analyze cEVs microRNAs in serum of advanced stage NSCLC patients with PD-L1 expression ≥50% at diagnosis, before first-line pembrolizumab, to evaluate their possible role as potential biomarkers for immunotherapy response prediction and outcomes.

**Methods:**

cEVs were isolated from serum of healthy subjects and NSCLC patients at diagnosis. All patients had tumor PD-L1≥50% and cEVs were extracted before first-line pembrolizumab treatment. cEVs were then characterized for morphology, integrity, concentration, size and protein contaminants. Subsequently, microRNA content (miR-10a, miR-21, miR-22, miR-30a, miR-34a, miR-106b, miR-125b, miR-150, miR-155, miR-181a, miR-181b, miR-451a) was investigated by digital PCR. Additionally, miRNA-targets and their roles were evaluated. All data were associated with immunotherapy response, Progression Free Survival (PFS), Overall Survival (OS), Eastern Cooperative Oncology Group Performance Status (ECOG-PS) and metastases.

**Results:**

Twelve NSCLC-related microRNAs have been found, for the first time, in serum cEVs from a specific cohort of metastatic advanced stage NSCLC patients. Through a functional analysis, these microRNAs are found to be connected to each other and involved in the pathology of NSCLC, particularly in IGF/P53/VEGF/NOTCH/PI3K pathways, in cytokine/interleukin signaling and in the immune system. Specifically, we demonstrated that cEV miR-106b, miR-451a, miR-181 and miR-10a were significantly up-regulated in non-responder patients compared to responder ones (*p*-value=0.08-0.1) predicting with high accuracy, already at diagnosis, treatment response. Furthermore, a low level of all these microRNAs predicted improved PFS (*p*-value=0.009-0.02) and a low amount of miR-106b predicted longer OS (*p*=0.069). In addition, it was observed that high levels of miR-106b and miR-451a are indicative of a high number of metastases (*p*=0.05/0.04, respectively) and of ECOG-PS=0.

**Discussion:**

This is the first study that investigated specific potential serum cEV miRNAs to predict with high accuracy immunotherapy response and prognosis in specific metastatic NSCLC patients, already at diagnosis. Collectively, our cEV miRNA analysis identifies novel circulating biomarkers that are easily accessible and non-invasive, offering a potential blood-based tool to guide personalized medicine in NSCLC.

## 1 Introduction

Lung cancer is the second most diagnosed cancer (11.4% of total cases), but the leading cause of cancer death (18% of the total cancer deaths) (1). It can be histologically classified into Small Cell Lung Cancer (SCLC) and Non-Small Cell Lung Cancer (NSCLC); this last is further classified into adenocarcinoma, squamous cell and large cell carcinoma (2, 3). Unfortunately, only a small proportion of NSCLC patients (<20%) are diagnosed at the early stage of the disease. Contrarily, most NSCLC patients are still diagnosed at later stages (IIIB/IV), when the tumor has already spread to multiple lymph nodes and/or to distant organs, negatively impacting the median survival (4). These last patients are generally treated with targeted therapy, alone or in combination with chemotherapy (5). Immune checkpoint inhibitors (ICIs), especially monoclonal antibodies that target PD-1 or its ligand (PD-L1), including pembrolizumab, restoring the antitumor immune response, revolutionized advanced stage NSCLC treatment strategies for enhancing survival (5). Even if this therapy prolongs survival and improves prognosis, unfortunately, some patients develop immune-related adverse events and other ones even do not respond (6). Predictive biomarkers, like microsatellite instability-high and tumor mutational burden (TMB) which correspond to somatic mutations, help to identify patients likely to respond to immunotherapy. An increased number of mutations results in higher neoantigen production and thus potentially increased immune recognition and response. Some studies reported that higher TMB is associated with improved outcomes. Intra-tumoral heterogeneity (ITH) can also affect immune response. Patients with high neoantigen burden and low ITH treated with ICIs had improved OS compared with those with a high ITH. Decreased T-cell infiltration has been reported to be associated with a poorer prognosis and to be predictive of a decreased response to ICIs (7). For these predictive biomarkers persist challenges in standardization and testing (8).

Although tumor-based PD-L1 expression remains the main immune-based biomarker in clinical practice for the ICI therapy, some patients with high PD-L1 expression do not benefit from ICI treatment as well as patients with low or negative PD-L1 expression, thus supporting the need to identify other reliable biomarkers (9). Of note that the use of tumor-based biopsy -biomarkers fails to capture both molecular and spatial heterogeneity of tumor and the dynamic tumor-host relationship. This limitation could be overcome by analysis of circulating biomarkers, which can reflect the systemic response of the tumor and allow repeated sampling and monitoring, and, above all, it is a minimally invasive diagnostic procedure for the patients.

Nowadays, Extracellular Vesicle (EV)-based liquid biopsy has emerged as an innovative and non-invasive approach in cancer diagnostics allowing them to overcome conventional tumor biopsy limitations (9). EVs are small lipid bilayer particles with a diameter range of 30nm-1µm (10). They are released by all cell types during physiological and pathological processes; for this reason, they are identified as a novel crosstalk mechanism between tumor cells and microenvironment (11). EVs are present in all biological fluids and protect their content, including RNAs, DNA, proteins and metabolites, from degradation. Recent studies evaluated EVs and their cargo as novel non-invasive biomarkers in several tumors, including lung cancer (12, 13). In particular, an important class of small (19–22 nucleotides) single-stranded non-coding RNAs, microRNAs (miRNAs/miR-), has emerged as key players in modulating cancer cell phenotype and in regulating innate/adaptative immune response by reducing the expression of key regulators of developmental checkpoints. For these reasons, many researchers studied both free circulating and EV-associated miRNA role as predictors of clinical outcome in ICI treated NSCLC patients (13–15).

Given the different roles that miRNAs have in cancer and its biogenesis, we hypothesized that some of the miRNAs associated with NSCLC (in tumor tissue and as free form in circulation) could also play a role in the response to therapy and specifically could be associated with prognosis and response to first-line therapy with pembrolizumab in NSCLC patients with PD-L1≥50%. Therefore, in this context, we selected twelve miRNAs, including miR-10a, miR-21, miR-22, miR-30a, miR-34a, miR-106b, miR-125b, miR-150, miR-155, miR-181a, miR-181b and miR-451a, and studied them in a specific cohort of NSCLC patients. To our knowledge, none of these miRNAs has been associated with a specific response to pembrolizumab. This study was conducted on serum because, both from a practical and clinical point of view, it represents the source sample of all analytes and markers used in clinical routine. Furthermore, from a technical point of view, serum is simple to obtain and is stable because it does not clot.

In our study we selected newly advanced NSCLC patients with PD-L1 ≥ 50% who are candidates for first-line monotherapy with pembrolizumab, an anti-PD-1 monoclonal antibody. We investigated the presence of cEVs in serum of NSCLC patient cohort and, subsequently, the expression of cEV miRNAs and their possible role in immunotherapy response prediction and prognosis.

## 2 Materials and methods

### 2.1 Patients’ characteristics, healthy donors and serum samples

Twenty-one advanced stage NSCLC patients were recruited at diagnosis, from 2018 to 2021 at IRCCS CROB, before first-line pembrolizumab (monoclonal antibody anti-PD-1) treatment as monotherapy​. Patients’ follow-up was until March 2024. Following them over time, they were also classified based on treatment response, analyzing radiological evaluations at 6 months after therapy initiation according to RECIST criteria (16): patients with stable disease or partial/complete response were considered responders (R), while those with disease progression were non-responders (NR). Progression free survival (PFS) and overall survival (OS) were measured from the immunotherapy initiation to the first disease progression and death, respectively. Patients’ clinicopathological characteristics were described in **Table 1**.

**Table 1:** Clinicopathological characteristics of advanced stage NSCLC patients.

Characteristics	Overall (n = 21)
Sex	
Male	13
Female	8
Age	
Median	67
Range	47-80
Smoking history	
Smoker	16
Non-smoker	1
Unknown	4
ECOG PS	
0	5
1	11
2	5
Stage	
IIIB	1
IV	20
Histological type	
Adenocarcinoma	16
Squamous cell carcinoma	5
Number of metastatic sites	
Median	2
Range	1-4
Lung metastasis	
Yes	11
No	10
Liver metastasis	
Yes	4
No	17
Brain metastasis	
Yes	6
No	15
Bone metastasis	
Yes	7
No	14
Lymph node metastasis	
Yes	17
No	4
Other metastasis (adrenal glands)	
Yes	3
No	18
PD-L1 IHC expression	
TPS > 50%	21
KRAS mutation	
Unknown	19
Mutated (uncommon)	2
EGFR mutation	
Wild type	20
Mutated (exon 20)	1
First-line treatment	
Pembrolizumab	21
Response to treatment	
Responder	11
Non-responder	10

ECOG PS, Eastern Cooperative Oncology Group Performance Status score; PD-L1, programmed death-ligand 1; TPS, Tumor Proportion Score; ICH, immunohistochemical.

In addition, peripheral blood (PB) samples from twenty-one healthy subjects (HS), matched for age, gender and lifestyle, were collected. This study complied with the international Helsinki Declaration regulation for research on human subjects and was approved by Regional Ethical Committee (Prot. N 2021-0007329). Prior written informed consent was obtained from all patients and HS in accordance with the approved experimental protocol.

Three ml of PB were collected from each patient and HS. PB samples, drawn into Vacutainer SST II Advance tubes (Becton Dickinson, BD, Franklin, NJ, USA), were centrifuged at 974×g at 4°C for 10 min to obtain serum then stored at -80°C until use.

### 2.2 cEV isolation

cEV isolation was performed using a bench centrifuge (MicroCL 21R centrifuge, Thermo Fisher Scientific, Wilmington, DE, USA), as previously reported (17). Briefly, 500 μl of serum were thawed at room temperature and centrifuged at 200×g for 5 min at 4°C. Supernatant was than centrifuged at 14,300×g for 1 hour at 4°C. Resulting pellet was washed with 0.22 μm filtered phosphate-buffered saline (PBS) without calcium and magnesium salts (Gibco), centrifuged at 14,300×g for 1 hour at 4°C and resuspended in 500 μl of 0.02 μm filtered PBS. According to MISEV 2023 guidelines which invite to confirm the presence of EVs after isolation analyzing morphology, quantity, size and the absence of non-EV components, the presence of EVs in our samples were confirmed using subsequent methods.

### 2.3 Transmission electron microscopic analysis

Twenty microliters of cEV sample suspension were applied to a Pioloform-coated Nickel grid (200 mesh; TAAB Laboratories Equipment Ltd., Aldermaston, UK). The grid was floated for 2 min on the sample drop and rinsed on a 20 μl double distilled water drop. Negative staining was performed with 200 μl of 1% w/v UA-zero EM stain solution (Agar-Scientific Ltd., Stansted, United Kingdom). After draining off the excess staining solution, the specimen was examined in a Philips Morgagni 282D TEM, operating at 60 kV. Electron micrographs of negatively stained samples were photographed on Kodak electron microscope film 4489 (Kodak Company, Rochester, NY, USA).

### 2.4 Flow cytometric analysis

cEV samples from advanced NSCLC and healthy subjects, were acquired on DxFlex flow cytometer and analyzed by Kaluza C software (Beckman Coulter, Brea, CA, USA). Megamix-Plus FSC (Biocytex, France), a mix of fluorescent beads of varied diameters (0.1, 0.3, 0.5 and 0.9 µm) was used to set up instrument for physical parameters forward scatter, FSC, and side scatter, SSC (**Supplementary Figure S1**). All parameters, including FSC and SSC, were set up in logarithmic scale and height (H) parameter and threshold was set on SSC. EV samples were labeled with 5-carboxyfluorescein diacetate-succinimidyl ester (CFDA-SE, Sigma-Aldrich) as previously reported (17). To set carboxyfluorescein-succinimidyl ester (CFSE) positive EV gate, 0.02 µm filtered PBS and unstained EVs were used (**Supplementary Figure S1**). A total of 50,000 events were acquired at a low flow rate.

### 2.5 Nanoparticle tracking analysis

Concentration and size distribution of cEVs were defined by Nanoparticle Tracking Analysis (NTA) as previously reported (17), using NanoSight NS300 (Malvern Panalytical Ltd, UK). cEV samples were processed immediately after isolation. Five videos of 60 sec each per sample were obtained in light scattering mode as indicated in the manufacturer’s protocol. Data were processed using NTA 3.2 software. cEV concentration, mean, mode, D10 and D90 values were reported.

### 2.6 Serum contaminant analysis

The concentration of lipoproteins, apolipoproteins and proteins were measured in serum, cEV samples and supernatants of three advanced NSCLC patients and three HS, as previously reported (17).

All analyses were conducted on the automatic analyzer AU680 (Beckman Coulter) using LDL-cholesterol, HDL-cholesterol and TG reagent Kits, Apo A1 and Apo B reagent kits and Total Protein and Albumin reagent kits (Beckman Coulter).

### 2.7 cEV RNA isolation, quantification and reverse transcription

Total cEV RNA has been isolated according to Patent n. 102023000020943, then it was immediately quantified by QUIBIT 4.0 Fluorometer (Thermo Fisher Scientific) using Qubit microRNA Assay kit (Life Technologies). cDNA was synthesized using TaqMan Advanced miRNA cDNA Synthesis Kit (Applied Biosystems, Foster City, CA, USA), following manufacturer’s instructions, starting from a precise cEV-RNA amount.

### 2.8 cEV miRNA analysis

Twelve miRNAs, including miR-10a-5p, miR-21-5p, miR-22-5p, miR-30a-5p, miR-34a-5p, miR-106b-5p, miR-125b-5p, miR-150-5p, miR-155-5p, miR-181a-5p, miR-181b-5p and miR-451a, were selected by literature for their involvement in NCSLC and for their possible role as diagnostic and prognostic biomarkers in this tumor (12, 14, 18–43). Most of them have been described as both tissue-specific and as plasma/serum free circulating or EV-associated miRNAs in NSCLC. We quantified the expression levels of the above miRNAs by QX200 droplet digital PCR (ddPCR) system (Bio-Rad Laboratories, Hercules, CA, USA). This technology is an emerging method to quantify nucleic acids with high accuracy and sensitivity. It is able to perform an absolute quantification down to a minimum of 0.2 copies/µl of a specific miRNA. For each miRNA, 10 μl of the synthesized cDNA, suitably diluted (1:200 for miR-10a-5p, miR-22-5p, miR-34a-5p, miR-155-5p and miR-181b-5p; 1:1000 for miR-106b-5p, miR-125b-5p, miR-150-5p and miR-181a-5p; 1:10^4^ for miR-21-5p and miR-30a-5p; 1:10^5^ for miR-451a), were added to a 2⨰ ddPCR supermix for probe (Bio-Rad) and 1 μl 20⨰ TaqMan miRNA specific probe (assay ID 479241_mir for hsa-miR-10a-5p, assay ID 477987_mir for hsa-miR-22-5p, assay ID 478048_mir for hsa-miR-34a-5p, assay ID 483064_mir for hsa-miR-155-5p, 477857_mir for hsa-miR-181b-5p, assay ID 478412_mir for hsa-miR-106b-5p, assay ID 477885_mir for hsa-miR-125b-5p, assay ID 477918_mir for hsa-miR-150-5p, assay ID 477857_mir for hsa-miR-181a-5p, assay ID 477975_mir for hsa-miR-21-5p, 479448_mir for hsa-miR-30a-5p and assay ID 478107_mir for hsa-miR-451a, ThermoFisher Scientific) in 20 μl reaction mix.

Then, droplets were generated by loading a plastic cartridge containing a mix with 70 μl of Droplet Generation Oil into the Droplet Generator (Bio-Rad). Droplets generated from each sample were carefully transferred into a 96-well plate and PCR amplification was carried out on a thermal cycler. Cycling conditions were 95°C for 10 min, then 45 cycles of 95°C for 15 sec and 58°C for 1 min, and finally 98°C for 10 min and 4°C infinite hold. A ramping rate of 2°C/sec was used in every step. The plate was then read in the Droplet Reader and analyzed using the QuantaSoft TM version 1.7.4 software (Bio-Rad).

For each sample, n. copies/µl generated by software analysis was multiplied by the used cDNA dilution factor to obtain n. copies/µl reported in graphs.

### 2.9 Systematic analysis of cEV miRNAs

The analysis of putative target genes was performed using miRTargetLink 2.0. Specifically, this tool was used in unidirectional mode, selecting “Homo sapiens” as species and “Target-gene overlap between multiple microRNAs” as miRNA-centric search. All twelve miRNAs have been inserted. To obtain a more precise network, we selected “strong validated miRNA targets”. Following this selection the software automatically deleted miR-22.

Resulted miRNA target-genes were used for enrichment analysis by ShinyGO 0.77 [ShinyGO 0.77 (sdstate.edu)], selecting different pathway databases including Kyoto Encyclopedia of Genes and Genomes KEGG, Panther, Reactome and WikiPathways, with 0.05 FDR cut-off and 10–2000 pathway size.

### 2.10 Statistical analysis

Non-parametric, unpaired (Mann-Whitney test) Student’s *t*-test was used to analyze two group comparisons. Results are shown as median with range.

Receiver Operating Characteristic (ROC) analyses were performed to evaluate the diagnostic ability of miRNA analysis in discriminating the true state of subjects, finding the optimal cut-off values.

Survival curves for PFS and OS were performed using Kaplan-Meier analysis and compared by log-rank (Mantel-Cox) test. The Cox model, a regression method for survival data, provides an estimate hazard ratio (HR) and its confidence interval (CI). HR is a measure of how often a particular event happens in one group compared to how often it happens in another group, over time. We used the median expression of each miRNA to subdivide the NSCLC patients into two groups: those with low and those with high miRNA expression levels (**Supplementary Table S1**). One patient was excluded from the analysis of OS because the death was due to SARS-CoV-2 infection.

All statistical tests were bilateral and a p-value <0.05 was considered significant.

All tests were performed using GraphPad Prism 10.2.1 software.

## 3 Results

### 3.1 Clinicopathological characteristics of advanced stage NSCLC patient cohort

Twenty-one sera of patients with advanced stage (IIIB/IV) NSCLC at diagnosis, before any type of treatment, were collected for this study. The complete patient clinicopathological characteristics were reported in **Table 1**. Among patients, there were n=13 men and n=8 women, and their median age was 67 years, with a range of 47–80 years. Most patients (n=16) were smokers and only one non-smoker, defining this latter as a patient who had never smoked or had smoked fewer than 10 packs/years. In addition, classifying patients on the basis of Eastern Cooperative Oncology Group Performance Status (ECOG PS), most patients (n=11) had ECOG PS 1, instead both ECOG PS 0 and ECOG PS 2 had n=5 patients each. Among our cohort, adenocarcinoma was the most frequent histological type (n=16 patients) compared to squamous cell carcinoma (n=5 patients). All patients had different metastasis at diagnosis, with a median of n=2 metastatic sites (range 1-4). All patients showed high immunohistochemical tumor PD-L1 expression (greater than 50%); therefore, they were candidates for first-line treatment with monotherapy anti-PD-1 pembrolizumab. Following them over time, they were also classified based on treatment response, analyzing radiological evaluations at 6 months after therapy initiation: patients with stable disease or partial/complete response were considered responders (R), while those with disease progression were non-responders (NR). In our patient cohort, we counted n=11 R and n=10 NR.

### 3.2 Characterization of cEVs from healthy subjects and advanced stage NSCLC patients

**Figure 1:**
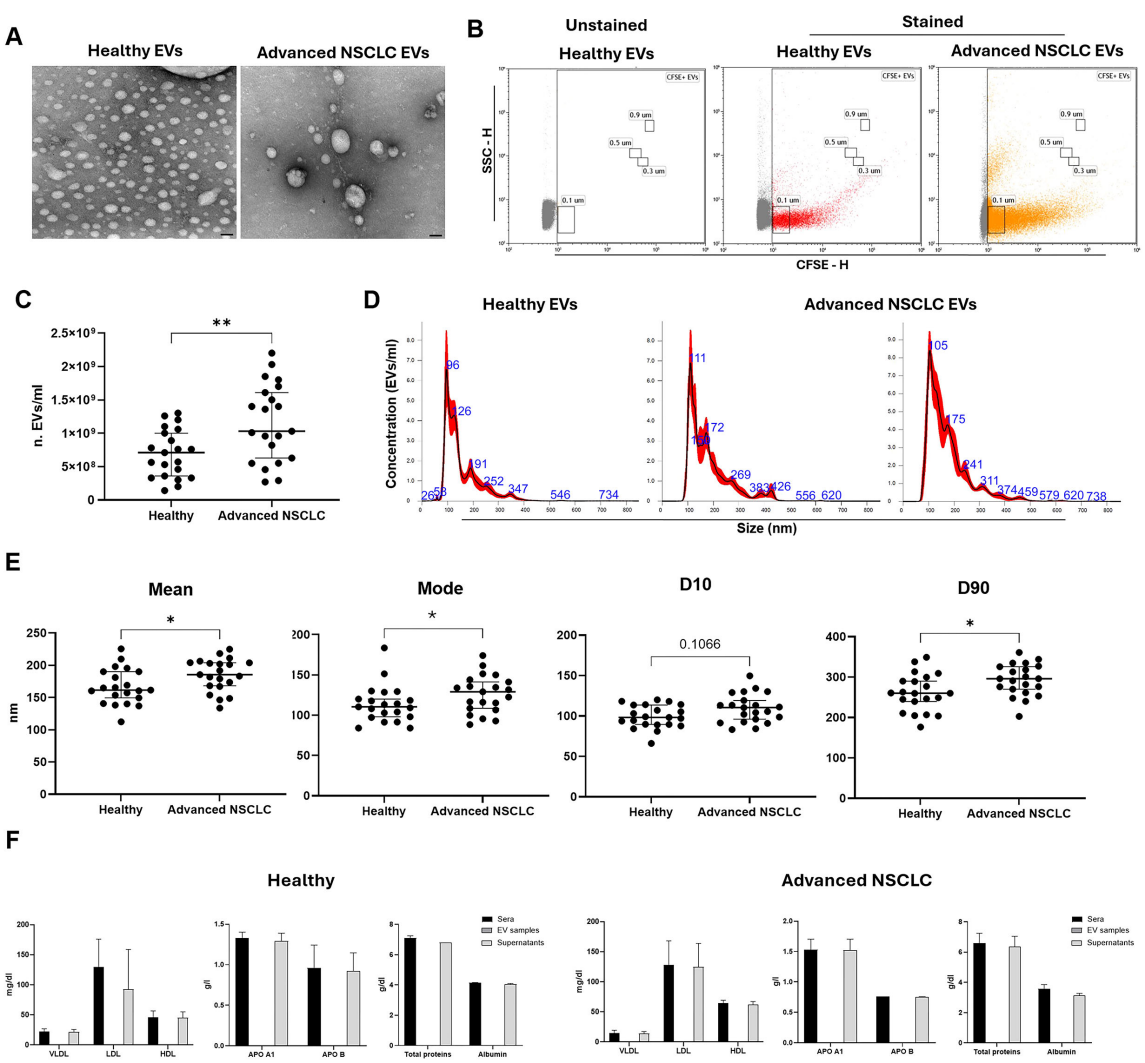
Characterization of cEVs from healthy subjects (HS) and advanced stage NSCLC patients. **(A)** Representative TEM photos of cEVs derived from HS and Advanced NSCLC patient (bars indicate 50 nm). **(B)** Flow cytometer CFSE-H/SSC-H dot plots of a representative unstained and stained HS EVs and stained NSCLC EVs. **(C)** NTA dot plot of cEV concentration (n. EVs/ml) comparison between n=21 HS vs n=21 advanced NSCLC patients. Each symbol represents single sample and horizontal bars represent median values. Statistically significant analyses are indicated by asterisks: **p < 0.01. **(D)** Three representative NTA size distribution profile of cEVs from one HS and two NSCLC patients. **(E)** NTA dot plots of cEV mean, mode, D10 and D90 of comparison between n=21 HS vs n=21 Advanced NSCLC patients. Each symbol represents single sample and horizontal bars represent median values. Statistically significant analyses are indicated by asterisks: *p < 0.05. **(F)** Column bar of lipoproteins (VLDL, LDL and HDL), apolipoproteins (APO A1 and APO B), total proteins and Albumin in sera, EV samples and supernatants of n=3 representative HS and NSCLC patients. The EV column bar is zero. The bar-graphs represent mean + SD from three independent experiments.

Using our previously established protocol to isolate cEVs from serum, we collected vesicles from n=21 HS and n=21 advanced stage NSCLC patients. Serum pellet characterization is needed to verify the presence of EVs, in terms of morphology, quantity and size, and the absence of non-EV components.Firstly, to evaluate cEV morphology, TEM analysis was performed on representative cEV samples. As shown in **Figure 1A**, EV pellets from HS and advanced NSCLC contained round particles with a double layer membrane. To confirm the integrity of bilayer membrane particles, cEVs from representative HS and NSCLC patient samples were analyzed by flow cytometer, properly set to detect small particles. **Figure 1B** showed that both HS and NSCLC pellets were enriched with CFSE positive particles, demonstrating the presence of cEVs which have internalized a membrane-permeant non-fluorescent CFDA-SE and whose integrases converted it into fluorescent CFSE. Flow cytometric analysis, comparing unstained and stained samples, showed the presence of cEVs with diameter approximately from 100 nm to 900 nm both in HS and in advanced NSCLC patients (**Figure 1B**). The NTA quantification of particle number concentration and size (in diameter) of cEVs from serum of all samples were reported in **Figures 1C–E**. Interestingly, cEV concentration was significantly higher in advanced NSCLC patients compared to HS (median of 10.3×10^8^ cEVs/ml vs 7.1×10^8^ cEVs/ml, p=0.009; **Figure 1C**). Size distribution showed that HS and NSCLC cEVs had a heterogenous diameter range between 26 and 734 nm with a large enrichment in small cEVs (**Figure 1D**). In particular, NTA size parameters, such as mean, mode and D90, were significantly higher in cEVs from NSCLC patients compared to those from HS (**Figure 1E**). Specifically, median values of mean were 185.6 nm in NSCLC vs 161.5 nm in HS (p=0.02), median of mode were 129 nm vs 110.4 nm (p=0.05), respectively, and median of D90 were 296 nm vs 260.3 nm (p=0.02), respectively. Instead, no significant difference was observed for D10 parameter (median values of 110.4 nm for NSCLC vs 98.3 nm for HS, p=0.1; **Figure 1E**).

Finally, we verified the presence of serum contaminants in cEV pellets, dosing VLDL, LDL, HDL APO A1, APO B, total proteins and albumin in sera, in cEVs samples and in its respective supernatants after isolation. No serum contaminants were detected in cEVs samples, while their amounts were comparable between that in the original serum and that in the remaining supernatant, in both HS and NSCLC samples (**Figure 1F**).

Collectively, using TEM, flow cytometry, NTA characterization and proteins’ assay, we confirmed the successful isolation of serum cEVs free from protein serum contaminants.

### 3.3 Analysis of miRNA content in advanced stage NSCLC cEVs

A total of twelve miRNAs were selected for their involvement in NSCLC and were evaluated to investigate their presence and role in cEVs of patient cohort (**Table 2**).

**Table 2:** Summary of selected miRNAs and their role in NSCLC.

miRNA	Sample type	Role	Ref.
miR-10a	tissue, serum	diagnostic; prognostic: associated with therapy response, clinical stage and metastasis	(14, 18, 19)
miR-21	tissue, plasma, serum, plasma cEVs, serum cEVs	diagnostic; associated with tumor size and stages; prognostic: associated with OS, recurrence and DFS, PFS and therapy response; involved in metastases	(12, 14, 20, 21, 44)
miR-22	tissue, plasma, serum	prognostic: associated with therapy response; involved in metastases	(12, 22, 23)
miR-30a	tissue, plasma cEVs	diagnostic	(24, 25)
miR-34a	tissue, plasma, serum	prognostic: associated with therapy response, OS and relapse; involved in metastases	(14, 26–28)
miR-106b	tissue, serum, plasma cEVs	prognostic: associated with therapy response, PFS and OS	(14, 29, 30)
miR-125b	serum, plasma cEVs	diagnostic; prognostic: associated with therapy response	(31, 32)
miR-150	tissue, plasma, serum cEVs	diagnostic; associated with histopathological evaluation and stage; prognostic: associated with DFS, PFS and OS; involved in metastases	(33–35)
miR-155	plasma, serum, serum cEVs	diagnostic; prognostic: associated with therapy response; involved in metastases	(14, 28, 36)
miR-181a	tissue, plasma, serum, plasma cEVs	prognostic: associated with therapy response, PFS, OS	(14, 37, 38)
miR-181b	tissue, plasma cEVs, serum cEVs	diagnostic; prognostic: associated with OS and DFS	(24, 39–41)
miR-451a	tissue, plasma, plasma cEVs	diagnostic; associated with stage; prognostic: associated with aggressive disease, vascular invasion, OS and DFS; involved in metastases	(41–43)

OS, Overall Survival; DFS, Disease Free Survival; PFS, Progression Free Survival.

**Figure 2:**
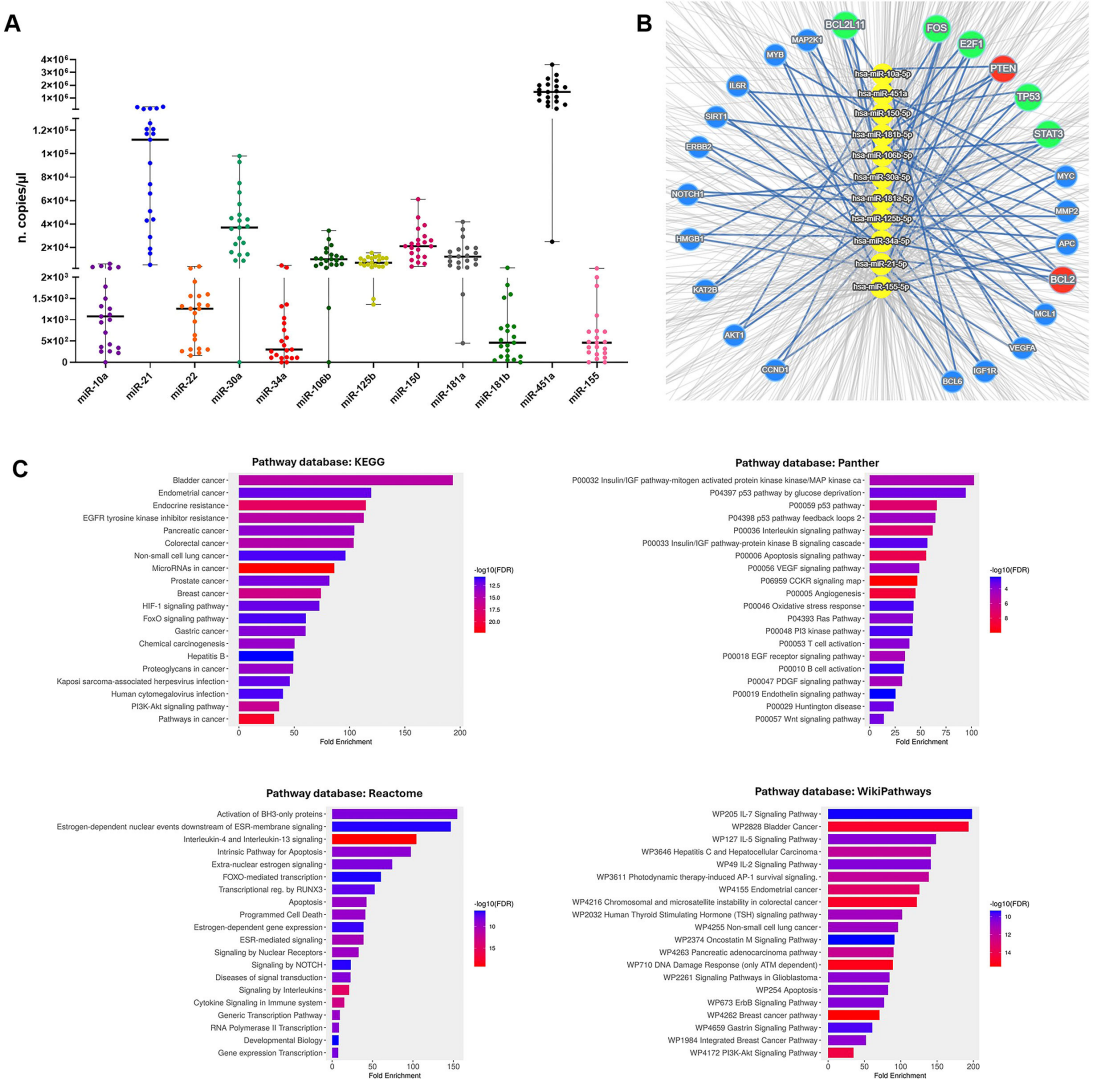
cEV miRNA detection in advanced NSCLC patients and systematic analysis of their targets. **(A)** Dot plots of miR-10a, miR-21, miR-22, miR-30a, miR-34a, miR-106b, miR-125b, miR-150, miR-155, miR-181a, miR-181b and miR-451a expressions in cEVs (n. copies/µl) from n=21 advanced stage NSCLC patients. Each symbol represents single sample and horizontal bars indicate the median values with range. **(B)** Concentric network layout obtained from miRTargetLink 2.0 indicating only strong-validated target-genes of microRNAs: miR-10a, miR-21, miR-30a, miR-34a, miR-106b, miR-125b, miR-150, miR-155, miR-181a, miR-181b and miR-451a. The target genes of six miRNAs are indicated in the red circle, the target genes of four miRNAs are indicated in the green circles and in blue circles the target genes of three miRNAs. **(C)** Pathway analysis of miRNA target-genes using KEGG, Panther, Reactome and WikiPathways databases.

Particularly, miR-10a, miR-21, miR-22, miR-30a, miR-34a, miR-106b, miR-125b, miR-150, miR-155, miR-181a, miR-181b and miR-451a levels were quantified within cEVs. As shown in **Figure 2**, all selected miRNAs were detected in NSCLC cEVs and they showed different expression levels. Among these, miR-451a, miR-21 and miR-30a were present in great quantity respect other ones (median 14.8x10^5^, 1.12x10^5^ and 3.7x10^4^ copies/µl, respectively).

We considered the possibility that these EV miRNAs mediate cell-to-cell communications supporting NSCLC disease. Therefore, we looked for their potential RNA targets by querying miRTargetLink 2.0 platform and identified 1387 targets (**Supplementary Table S2**). To perform a stringent analysis, we focused on 24 targets which were common for more miRNAs (**Figure 2B**; **Supplementary Table S3**). Specifically, miR-21, miR-34a, miR-125b, miR-181a/b and miR-451a targeted together BCL2, while miR-10a, miR-21, miR-106b, miR-155 and miR-181a/b targeted PTEN. Moreover, the combination of four miRNAs targeted FOS, TP53 and STAT3, and the combination of three miRNAs targeted other mRNAs (**Supplementary Table S3**).

Gene ontology enrichment analysis of these 24 targets showed “Cancer”, including NSCLC, as the most highly enriched KEGG term (**Figure 2C**). Panther and Reactome signaling maps revealed that almost all targets converged in the IGF, P53, VEGF, NOTCH and PI3k pathways (**Figure 2C**). In addition, their involvement in the signaling of interleukins, including IL-4, IL-13, IL-5, IL-2, IL-7, and in immune cell regulation, such as T and B cells, were identified in Reactome and WikiPathways databases (**Figure 2C**).

### 3.4 Association of cEVs and their miRNA content with immunotherapy response, survival outcomes and clinicopathological characteristics in advanced stage NSCLC patients

To understand if cEVs and their miRNA content analyzed in serum of advanced stage NSCLC patients at diagnosis, before any type of treatment, were able to predict immunotherapy response, we examined their levels stratifying our NSCLC patient cohort in R and NR.

Particle number and size did not discriminate between R and NR patients in a significative manner (**Supplementary Figure S2A**).

**Figure 3:**
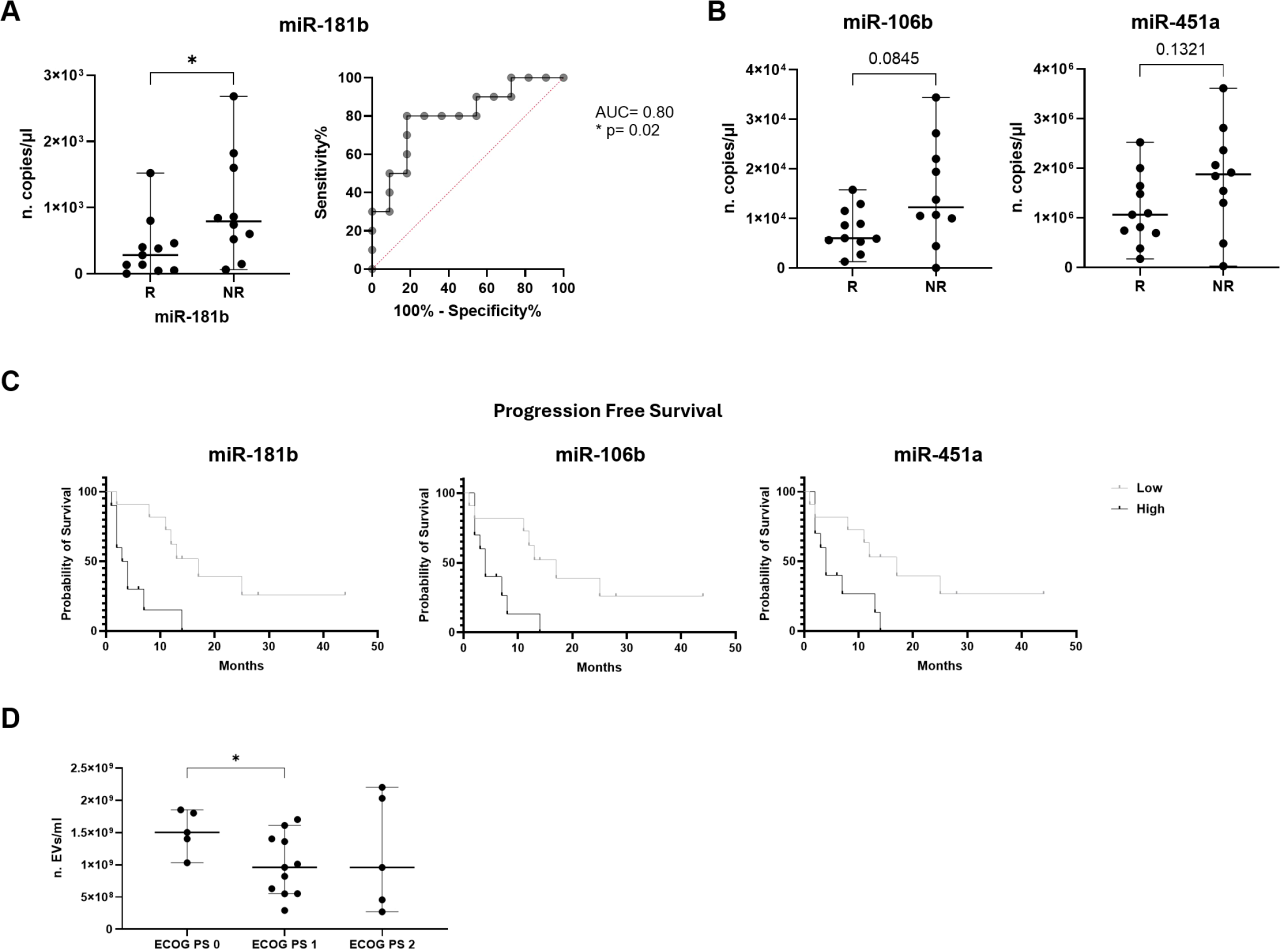
Association of miRNA expression with immunotherapy response and survival outcomes in advanced NSCLC patients. **(A)** Dot plot comparison and ROC curve of miR-181b expression (n. copies/µl) in responder (R) patients vs non-responder (NR) ones. **(B)** Dot plots comparison of miR-106b and miR-451a expression (n. copies/µl) in R vs NR. The horizontal bars indicate the median values with range. AUC and statistical analysis (p-value and/or asterisks *p<0.05) are indicated on graphs. **(C)** Kaplan-Meier curves for PFS of cEV miR-181b, miR-106b and miR-451a in advanced NSCLC patients. Median expression values classified patients into low/high expression groups. Log-rank (Mantle-Cox) test was used to compare two curves. **(D)** Dot plot comparison of cEV concentration (EVs/ml) in advanced NSCLC patients divided for ECOG PS scale. Horizontal bars represent median values. Statistically significant analyses are indicated by asterisks: *p < 0.05.

Regarding cEV miRNA content, of note, miR-181b expression showed a statistically significant difference between the two groups. In particular, NR patients showed a higher miR-181b level compared to R ones (median value of 790 vs 280 copies/µl, respectively; p=0.02; **Figure 3A**). In addition, to evaluate the discriminatory efficacy of this miRNA level in distinguishing NR and R patients before immunotherapy treatment, ROC analysis was applied. Interestingly, it established a cut-off value >490 copies/µl with 80% Sen and 81.82% Spe, showing an AUC of 0.8 (p=0.02; **Figure 3A**). Moreover, a similar trend was observed for miR-106b and miR-451a, which levels resulted higher in NR compared to R (median value of 12.25x10^3^ vs 6x10^3^, p=0.08, and of 1.88x10^6^ vs 1.06x10^6^, p=0.1, respectively; **Figure 3B**).

The expression of the remaining miRNAs was not significantly different in the two comparison groups (**Supplementary Figure S2B**).

To assess possibly prognostic values, cEVs and their miRNA levels were correlated with PFS and OS in all advanced stage NSCLC patients.

EV concentration and size did not correlate with PFS and OS (**Supplementary Figure S3A**).

On the contrary, as shown in **Figure 3C**, the Kaplan-Meier curve indicated that advanced stage NSCLC patients at diagnosis with low miR-181b, miR-106b and miR-451a expression had a significantly longer PFS compared to those with high miRNA expression (median PFS: 17 vs 3.5 months, p=0.0016 for miR-181b; median PFS: 17 vs 4 months, p=0.007 for miR-106b and median PFS: 17 vs 4 months, p=0.02 for miR-451a). Furthermore, in **Table 3**, it was reported that miR-181b had HR of 0.2688 which indicated that a low expression of this miRNA had a low-risk rate of progression (26.88%) compared to one with higher expression (73.12%). Instead, miR-106b and miR-451a had HR of 0.3216 and 0.3585, respectively.

**Table 3:** Log-rank (Mantle-Cox) tests for PFS and OS in n=21 and n=20 advanced stage NSCLC patients, respectively.

Variable	PFS				OS			
	HR (95% CI)	*p* Value	Median Survival		HR (95% CI)	*p* Value	Median Survival	
			*Low*	*High*			*Low*	*High*
miR-10a	0.6965 (0.2496-1.944)	0.43	12	5.5	1.173 (0.3902-3.524)	0.77	14	18
miR-21	0.9815 (0.3651-2.639)	0.97	12	7	0.8060 (0.2667-2.435)	0.69	18	10
miR-22	0.7712 (0.2803-2.122)	0.59	12	7	1.249 (0.4178-3.737)	0.69	14	18
miR-30a	0.7522 (0.2725-2.076)	0.55	13	5.5	1.252 (0.4187-3.744)	0.68	18	10
miR-34a	0.6649 (0.2445-1.808)	0.39	12	5.5	0.5425 (0.1777-1.656)	0.25	18	8
**miR-106b**	**0.3216** **(0.1095-0.9442)**	**0.007**	**17**	**4**	0.5418 (0.1775-1.654)	0.25	29	8
miR-125b	0.8091 (0.2957-2.213)	0.66	13	5.5	1.453 (0.4894-4.316)	0.5	16	37.5
miR-150	0.9902 (0.3685-2.661)	0.98	11	8.5	0.8641 (0.2878-2.595)	0.79	29	14
miR-155	0.5938 (0.2209-1.596)	0.28	12	5.5	0.4545 (0.1511-1.367)	0.14	30	9
miR-181a	1.007 (0.3751-2.702)	0.99	12	5.5	1.686 (0.5683-5.003)	0.34	13.5	29
**miR-181b**	**0.2688** **(0.0876-0.8252)**	**0.0016**	**17**	**3.5**	0.48 (0.1366-1.405)	0.17	29	8
**miR-451a**	**0.3585** **(0.1249-1.029)**	**0.02**	**17**	**4**	0.5765 (0.1901-1.746)	0.30	29	8

PFS, Progression Free Survival; OS, Overall Survival; HR, Hazard Ratio (low/high); CI, Confidence Intervals. Patients were classified into low or high groups according to the median expression value of each miRNA. In bold are indicated the significative values.

No association between PFS and other miRNAs was observed in all advanced NSCLC patients (**Table 3**; **Supplementary Figure S3B**). Regarding patient mortality of the entire advanced stage NSCLC patient cohort, it was observed that miRNAs were not associated with OS (**Table 3**; **Supplementary Figure S3B**).

**Figure 4:**
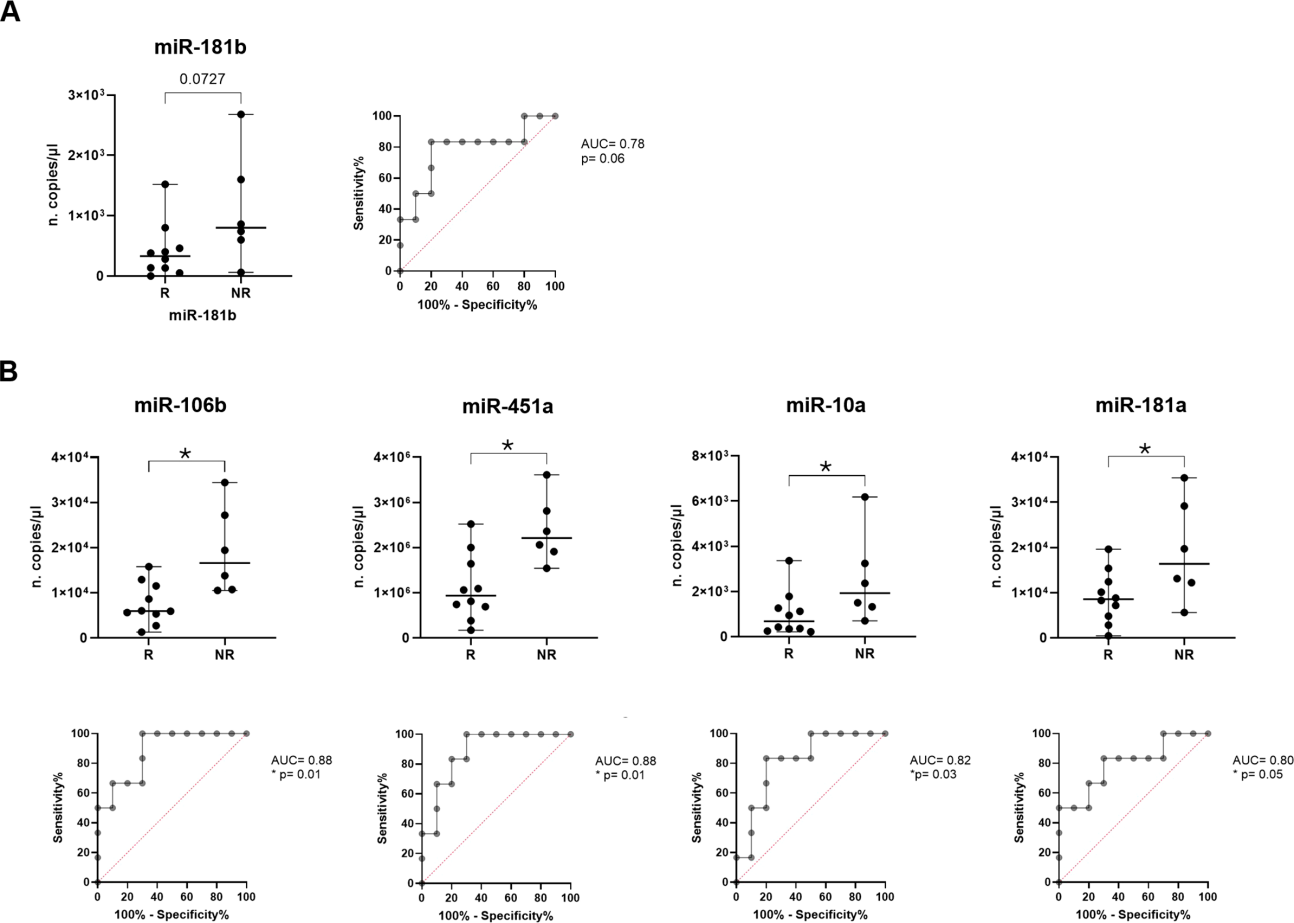
Analysis of miRNA expression in R vs NR advanced adenocarcinoma NSCLC patients. Dot plot comparison and ROC curve of **(A)** miR-181b **(B)** miR-106b, miR-451a, miR-10a and miR-181a expression (n. copies/µl) in R vs NR adenocarcinoma NSCLC patients. The horizontal bars indicate the median values with range. AUC and statistical analysis (p-value and/or asterisks *p<0.05) are indicated on graphs.

Subsequently, we wanted to verify the possible association between cEV amount and cEV miRNA levels with NSCLC clinicopathological characteristics, such as histological type, metastasis, smoking history and ECOG PS. Firstly, as regards the histological subtype, adenocarcinoma or squamous cell carcinoma, none of the above mentioned cEVs parameters is able to discriminate between the two subtypes. (**Supplementary Figures S4A, B**). For number of metastatic sites, the entire patient cohort was subdivided into two groups, the first one included patients with n=1/2 metastatic sites, the second one included patients with n=3/4 metastatic sites. There are no significant differences between the two groups of patients neither for the concentration, nor for the size, nor for the miRNAs, even if some miRNAs such as miR-34a, miR-106b, miR-125b and miR-451a, show higher levels high in expression in patients with n=3/4 metastatic sites compared to patients with n=1/2 metastatic sites (**Figures 4A, B**).

In addition, EV concentration was correlated with ECOG PS scale showing a significant difference between cEVs amount in patients with ECOG PS=0 and ECOG PS=1 (p=0.03, **Figure 3D**). No other significant data for ECOG PS were observed for EV number, size and miRNA content (data not shown).

### 3.5 Association of cEV miRNAs with immunotherapy response, survival outcomes and clinicopathological characteristics in adenocarcinoma patient subgroup

Since most of the patients in our cohort belong to the adenocarcinoma histological subtype, we restrict all the analysis to the 16 adenocarcinoma patient samples.

Of note, as regards the immunotherapy response prediction by restricting the cohort to adenocarcinoma patients, miR-181b confirmed its characteristic of being able to discriminate between two groups, showing a higher median value in NR vs R (800 vs 330 copies/µl, respectively, p=0.07; **Figure 4A**). Interestingly, it was observed that expression of miR-106b, miR-451a, miR-10a and miR-181a resulted significantly higher in NR compared to R (median of 16.6x10^3^ vs 5.95x10^3^, p=0.01 for miR-106b; median of 22.1x10^5^ vs 9.35x10^5^, p=0.01 for miR-451a; median of 19.3x10^2^ vs 6.8x10^2^ copies/µl, p=0.04 for miR-10a and median of 16.4x10^3^ vs 8.55x10^3^, p=0.05 for miR-181a; **Figure 4B**).

For all these miRNAs, ROC analyses were applied obtaining cut off value of >530 for miR-181b (Sen: 83.33%, Spe: 80%) with AUC of 0.78 (p=0.06), of >9.55x10^3^ for miR-106b (Sen: 100%, Spe: 70%) with AUC of 0.88 (p=0.01), of >13.15x10^5^ for miR-451a (Sen: 100%, Spe: 70%) with AUC of 0.88 (p=0.01), of >5.6x10^2^ (Sen: 100%, Spe: 50%) for miR-10a with AUC of 0.82 (p=0.03) and of >11.15x10^3^ for miR-181a (Sen: 83.33%, Spe: 70%) with AUC of 0.8 (p=0.05) (**Figure 4B**).

About other miRNAs, miR-30a showed an interesting trend resulting higher in NR vs R (p=0.07); instead, there were no statistically significant differences in miR-21, miR-22, miR-34a, miR-125b, miR-150 and miR-155 expression between two patient groups (**Supplementary Figure S6**).

**Figure 5:**
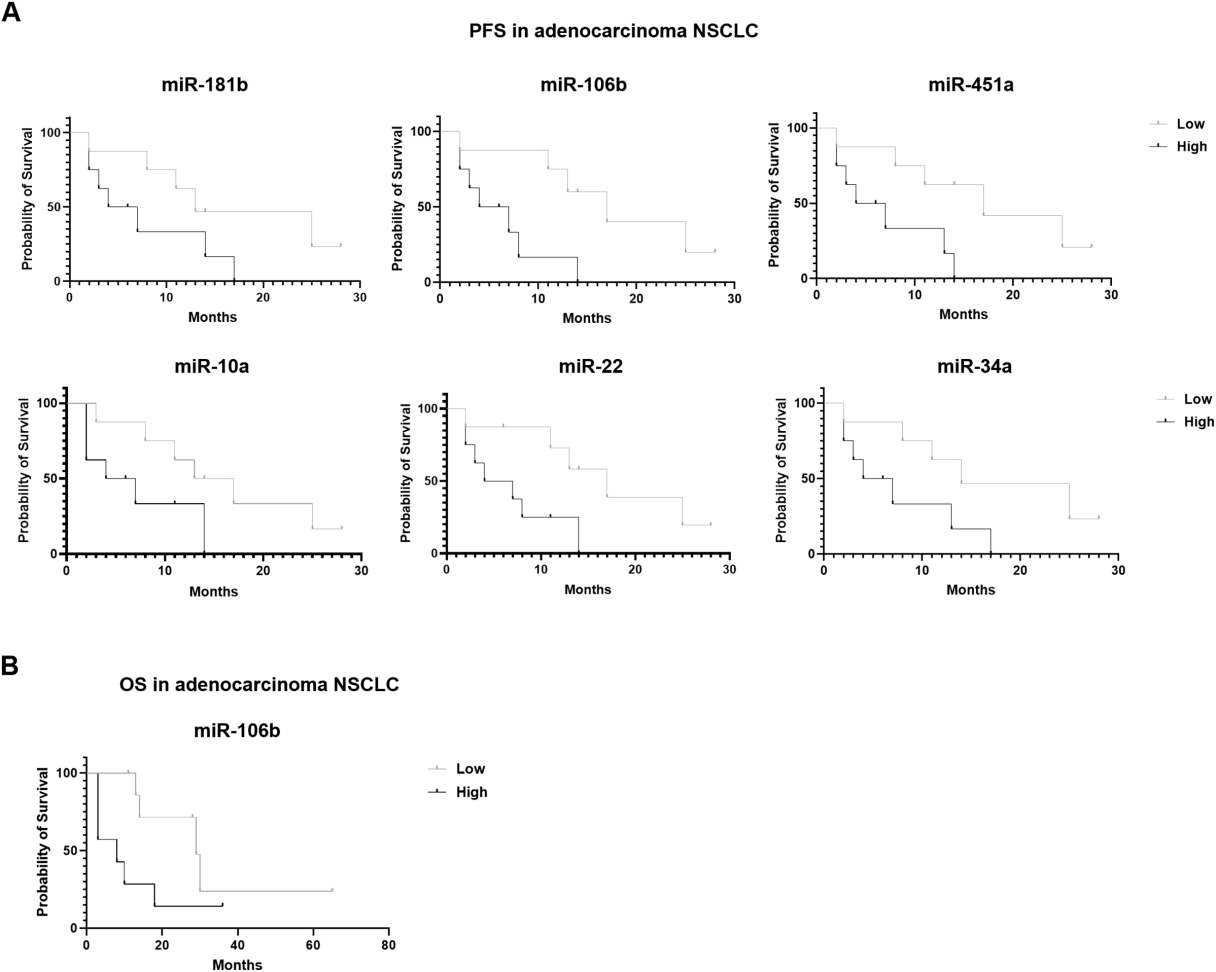
Association of miRNA expression with survival outcomes in advanced stage NSCLC patients. Kaplan-Meier curves for **(A)** PFS according to the expression of cEV miR-181b, miR-106b, miR-451a, miR-10a, miR-22 and miR-34a, and for **(B)** OS according to the expression of cEV miR-106b in adenocarcinoma NSCLC patients. Median expression values classified patients into low/high expression groups. Log-rank (Mantle-Cox) test was used to compare two curves.

Regarding survival outcomes, in adenocarcinoma context, six miRNAs showed a correlation with PFS and only one with OS. In particular, higher expressions of miR-181b, (p=0.07) miR-106b (p=0.009), miR-451a (p=0.02), miR-10a (p=0.07), miR-22 (p=0.02) and miR-34a (p=0.05) resulted in a shorter PFS (**Figure 5A**). Detailed data of median PFS and HR were reported in **Table 4**.

**Table 4:** Log-rank (Mantle-Cox) tests for PFS and OS in n=16 and n=15 advanced stage adenocarcinoma NSCLC patients, respectively.

Variable	PFS				OS			
	HR (95% CI)	*p* Value	Median Survival		HR (95% CI)	*p* Value	Median Survival	
			*Low*	*High*			*Low*	*High*
**miR-10a**	**0.4019** **(0.115-1.404)**	**0.07**	**15**	**5.5**	1.255 (0.3615-4.356)	0.71	21.5	18
miR-21	0.5848 (0.181-1.889)	0.32	13	7	1.582 (0.4577-5.465)	0.45	14	29
**miR-22**	**0.3219** **(0.0948-1.093)**	**0.02**	**17**	**5.5**	0.9722 (0.2814-3.359)	0.96	29	14
miR-30a	0.5342 (0.1642-1.752)	0.23	13	7	1.48 (0.4283-5.111)	0.53	14	18
**miR-34a**	**0.3594** **(0.1084-1.192)**	**0.05**	**14**	**5.5**	0.4228 (0.1185-1.509)	0.15	30	8
**miR-106b**	**0.2815** **(0.801-0.9889)**	**0.009**	**17**	**5.5**	**0.3427** **(0.0926-1.269)**	**0.069**	**29**	**8**
miR-125b	0.5282 (0.1607-1.736)	0.21	14	7.5	1.529 (0.4427-5.283)	0.49	18	13
miR-150	0.8413 (0.2702-2.62)	0.75	9.5	13	0.9487 (0.2745-3.279)	0.93	13	18
miR-155	0.7446 (0.2401-2.309)	0.6	13	7.5	0.6664 (0.1929-2.302)	0.51	30	14
miR-181a	0.6114 (0.191-1.962)	0.36	13	5.5	1.772 (0.5108-6.147)	0.35	14	29
**miR-181b**	**0.387** **(0.118-1.266)**	**0.07**	**13**	**5.5**	1.087 (0.3143-3.758)	0.89	14	18
**miR-451a**	**0.3164** **(0.093-1.079)**	**0.02**	**17**	**5.5**	0.9199 (0.266-3.181)	0.89	29	14

PFS, Progression Free Survival; OS, Overall Survival; HR, Hazard Ratio (low/high); CI, Confidence Intervals. Patients were classified into low or high groups according to the median expression value of each miRNA. In bold are indicated the significative values.

Interestingly, miR-106b also emerged for its correlation with OS (**Figure 5B**). Specifically, adenocarcinoma patients at diagnosis with low expression of miR-106b had a significantly longer OS compared to patients who had a high expression (p=0.069; median OS of 29 vs 8 months, respectively). The HR of 0.3427 indicates a low-risk rate of death with a low level of miRNA expression compared to a higher expression (**Table 4**).

The expression of remaining miRNAs was not significantly associated with PFS and OS (**Table 4**; **Supplementary Figure S7**).

**Figure 6:**
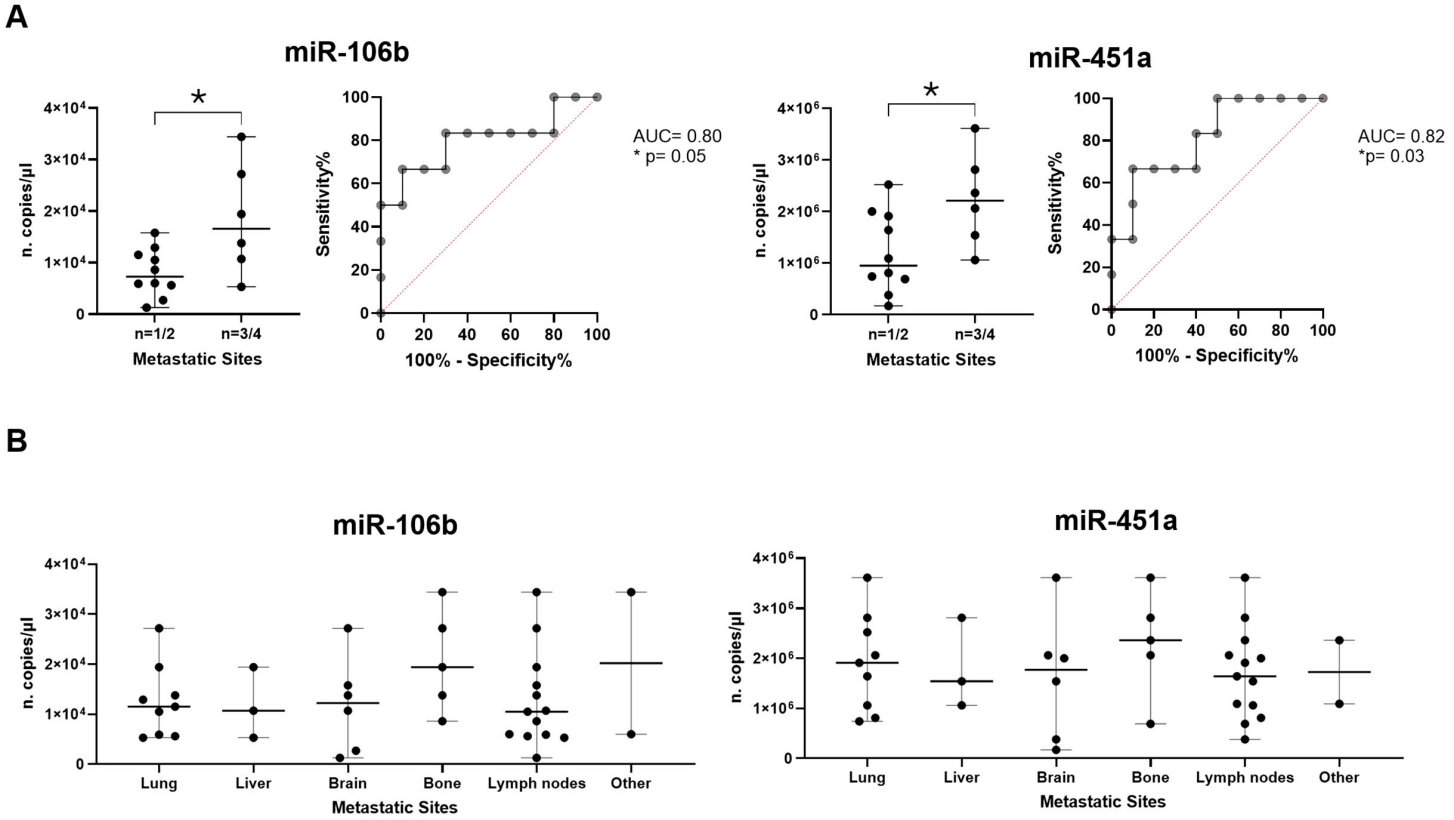
Association of miRNA expressions and metastases. **(A)** Dot plot comparisons and relative ROC curves of miR-106b and miR-451a expression (n. copies/µl) in patients with n=1/2 metastatic sites vs patients with n=3/4 metastatic sites. **(B)** Dot plots of miR-106b and miR-451a expression in different metastatic sites (lung, liver, brain, bone, lymph nodes and others). The horizontal bars indicate the median values with range. AUC and statistical analysis (p-value and/or asterisks *p<0.05) are indicated on graphs.

Subsequently, in adenocarcinoma context, we verified if miRNA expressions were able to distinguish patients with n=1/2 metastatic sites from n=3/4 ones. Interestingly, it was observed that miR-106b and miR-451a were significantly higher in patients with n=3/4 metastatic sites compared to patients with n=1/2 sites (median of 16.6x10^3^ vs 7.3x10^3^ copies/µl with p=0.05 and median of 22.1x10^5^ vs 9.5x10^5^ copies/µl with p=0.04, respectively; **Figure 6A**). ROC analysis to evaluate the discriminatory efficacy to distinguish n=3/4 to n=1/2 metastatic sites established a cut-off value of >10.6x10^3^ copies/µl (Sen: 83.33%, Spe: 70%) with AUC of 0.8 (p=0.05) for miR-106b and >9.35x10^5^ (Sen: 100%, Spe: 50%) with AUC 0.82 (p=0.03) for miR-451a (**Figure 6A**). Detailing miRNA distribution in metastasis type, we observed that both miRNAs were more abundant in patients with bone metastasis compared to other metastatic types (**Figure 6B**).

Other remaining miRNAs did not show significant differences between patients with n=1/2 metastatic sites compared to patients with n=3/4 sites (**Supplementary Figure S8**).

Finally, analyzing ECOG PS, miRNA levels were not associated with this score (data not shown).

## 4 Discussion

In the era of personalized medicine, the identification of circulating biomarkers easily accessible and non-invasive could be even more useful for response treatment prediction. cEVs have attracted much interest in cancer diagnosis and prognosis because they are abundant and stable in circulation, transport cellular information and have essential biological functions.

In our study, we reported data about serum cEVs and their miRNAs in a specific cohort of untreated patients with advanced stage (IIIB/IV) NSCLC at diagnosis, having PD-L1 immunohistochemical expression >50%, a median of two metastatic sites and eligible for first-line monotherapy with anti-PD-1, pembrolizumab. To our knowledge, this is the first study reporting this peculiar combination of serum cEVs and a specific NSCLC patient cohort.

In EV studies, it is necessary to carry out a complete and precise characterization of EVs isolated from biological fluids to proceed with the subsequent analyses. According to MISEV 2023, our workflow led us to isolate cEVs which are round, heterogeneous, enriched in small-sized EVs and, of note, free of serum contaminants. In particular, this last characteristic is important for the reliability of subsequent EV analysis, for example, by the exclusion of miRNA-associated with free proteins.

It emerged, from literature studies, that NSCLC cEV data were obtained from plasma and/or sera samples (**Table 2**). Noteworthy, in EV-based diagnosis tests of NSCLC, serum was mainly used to detect EV miRNAs (45), in fact it represents the actual source of circulating tumor biomarkers in clinical practice routine. Furthermore, the use of serum as cEV source associated with our easy cEV isolation and analysis, could be a great advantage in terms of immediate clinical practice translationality.

Regarding cEV concentration, we observed that NSCLC serum cEV amount was higher compared to healthy donors according to literature (20). This data could be due both to tumor burden and metastatic sites. To our knowledge, there was no data on EV concentration in healthy donors and non-metastatic NSCLC to do a comparison. Nevertheless, our data agrees with previous study that reported an increase of plasma EV concentration in metastatic NSCLC (46), while another study did not find such a difference in plasma of IIIB-IVA stage NSCLC patients (47). Of note, we observed a trend with a higher EV concentration in NSCLC with 3/4 metastatic sites compared to those with 1/2 ones (*p*=0.4). This data could point towards an additional effect of metastases on the EV concentration. Future studies are needed to better define it.

Moreover, all twelve miRNAs selected for their known role in NSCLC were found, with different amounts, in serum of our cohort of NSCLC patients. This data was very interesting, firstly because we confirmed the presence of these miRNAs, including miR-21, miR-30a, miR-125b, miR-150, miR-155, miR-181b and miR-451a, into cEVs despite the different *i*) starting source (serum instead of plasma), *ii)* isolation method (centrifuge instead of ultracentrifuge, kits, etc.) and *iii)* miRNA analysis (advanced RT-ddPCR instead of RT-qPCR) compared to those reported in other papers (**Table 2**). Secondly because we confirmed all miRNAs in our specific cohort, providing additional support to their association with NSCLC also in metastatic advanced stages. In addition, we pointed out the presence of other miRNAs, including miR-10a, miR-22, miR-34a, miR-106b and miR-181a, previously found only in tumor tissue and as circulating free form, into cEVs.

Our findings were original because we used a small volume of serum to obtain a heterogeneous population of cEVs. Additionally, we absolutely quantified EV-miRNAs using a precise amount of retrotranscribed EV-RNA. Moreover, these analyses led us to define EV-miRNAs as predictors of pembrolizumab response in a specific NSCLC patient cohort. Thus, our findings provide methodological and clinical novelty useful for clinical practice translationality. All this supported the goodness of our cEVs isolation-analysis procedure already applied to hematological malignancies ( (17) and Patent n. 102023000020943).

It is well known that EV-derived miRNAs could exert specific functions, in the context of lung cancer, also in terms of cell-to-cell communication both locally and at distance. In fact, all evaluated EV-miRNAs target genes were involved in different tumors and converged in specific pathways, such as IGF, P53, VEGF, NOTCH and PI3k pathways, in cytokine/interleukin signaling, including IL-4, and in immune regulation. Of note, all these pathways/signaling were found to be compromised in NSCLC (48–54). For example, recent studies have identified the role of the IGF axis and of dysregulation of downstream signaling molecules, including PI-3K/Akt and MAPK pathways, reporting that they jointly increase the risk of growth, invasion and metastasis in NSCLC (48, 52). Additionally, it was recently reported that IL-4 controls monocyte and macrophage immunosuppression in NSCLC, and that the relevant site of IL-4 signaling is not the tumor itself, but the bone marrow, where it drives pro tumorigenic myelopoiesis (53). Regarding the involvement of miRNA targets in the immune regulation which concerns both T and B cell activation, more interesting, it was observed that T cell dominated, and B cells were the second most common immune cell type in NSCLC (54). Furthermore, there are several proposed mechanisms for primary and secondary resistance to ICIs, including both intrinsic and extrinsic factors. For example, mutations in the IFN-y pathway, increased neoantigen intratumor heterogeneity, low mutational burden, transcriptomic features, epigenetic modifications, mutations in β2-microglobulin and deficiencies in HLA antigen presentation have been previously described as intrinsic to resistance to ICIs (55). Regarding P53, it is known that it led to immunotherapy primary resistance (56). Our data could add a new point of view regarding resistance to pembrolizumab in NSCLC by also turning attention to new EV mediated pathways. Certainly, functional studies are needed to confirm links between miRNAs and NSCLC pathways.

Notably, all these data led us to emphasize that cEVs and their miRNA content are released by all body cells which “suffer” the metastatic condition, suggesting that EV miRNAs cooperatively regulate the metastatic NSCLC and its communication with other cells, in particular with immune cells, locally and at distance sites.

Analyzing serum cEVs miRNA in advanced stage NSCLC patients, our data highlighted that miR-106b, miR-181b and miR-451a were able to discriminate between responders and non-responders to pembrolizumab with high accuracy. To our knowledge, there was no previous evidence showing these cEV-miRNAs and their relationship with immunotherapy response. Some information concerns their presence as free form in circulation. In particular, serum free miR-106 and miR-181 were found up-regulated in responders vs non-responders and, their expression in responders increased passing from pre- to post-therapy in multi-treated NSCLC patients before nivolumab therapy (14). Regarding cEVs and immunotherapy response, Peng et al. identified a cluster of plasma exosomal miRNAs (miR-320) which correlated with an unfavorable response to anti-PD-1 treatment in multi-treated NSCLC patients (31). All these supported our idea that cEVs and their miRNAs could be new biomarkers in immunotherapy response. Next investigations are needed to confirm it.

In addition, our cEV miR-106b, miR-181b and miR-451a were also able to make a PFS prognosis and, only for miR-106b, to make OS prognosis and to separate patients based on metastasis number.

Our result was in agreement with Xue et al. which found that high expression of a cluster of EV miRNAs, including miR-106b, was associated with a lower survival rate in lung adenocarcinoma (30). In particular, we found a HR value of 0.34 for OS; it means that NSCLC patients with low miR106b will have 66% of reduction of the mortality rate (i.e. time to mortality is slower), while those with higher miR106b levels will have 34% of increase in the mortality rate. HR indicates a risk rate of an event of interest in the next interval among individuals who have not yet experienced the event. Therefore, it is clinically relevant because it quantifies the effect of treatment (pembrolizumab, in this case) over time to a specific event such as dead. Overall, the quantification of EV-associated miRNAs could have a very important prognostic relevance in clinical oncology.

Concerning miR-181a/b, it was associated with a wide range of pathologies, including tumors, targeting key genes involved in evasion of growth suppression or resistance to cell death, and are regulators of angiogenesis, invasion and metastasis (57). Recently, Junliang Ma et al. reported that miR-181b was up-regulated in exosomes derived from NSCLC patients’ serum and cells (40). The expression levels of miR-181a/b in lung cancer are related to clinical-pathological characteristics such as patient survival rate, TNM staging and drug resistance (58). In particular, Yang et al. described poorer OS and DFS in NSCLC patients with low miR-181b expression in surgically removed tumor tissues (39), while in plasma cEVs it was identified as potential diagnostic biomarker for early-stage NSCLC (24), but never associated with survival (59). In agreement with us, recently, it was reported that plasma cEV miR-181, together with other miRNAs and combined with performance status, was capable of discriminating patients unlikely from those that are likely to benefit from immunotherapy (38). In this last paper, all data concern NSCLC patients who received nivolumab as second-line therapy unlike our study in which patients received it as first-line.

Regarding miR-451a which resulted in the most abundant miRNA in our NSCLC cEVs, it was reported that its low expression was related to shorter OS (43). Moreover, plasma exosomal miR-451a was identified as biomarker for the prediction of recurrence and prognosis in stage I-III NSCLC patients (42). Overall, miR-106b, miR-181 and miR-451a could represent a new signature for immunotherapy response and PFS/OS prognosis prediction in NSCLC and this could have a possible implication in the clinical setting. Further studies will be needed to verify our findings in additional patient samples, and to elucidate the mechanisms of action of these potential biomarkers in NSCLC.

Generally, the presence of metastasis in cancer patients indicates poor prognosis. The detection of both number and type of metastatic site through cEV analysis would be more advantageous because their incidence, at specific sites, worsens the general condition of these patients. In fact, it was abundantly demonstrated that survival rates for NSCLC patients with liver or brain metastases remain low, with overall poor outcomes and prognosis (60, 61). In fact, nearly 80% of patients with bone metastasis suffered from significant pain that compromises the quality of life. These patients developed skeletal-related events, like hypercalcemia, pathological fracture, spinal cord compression and others, which further decreased the patient’s survival time to 5.8-7 months (62). It is now well known that cEVs and miRNA expression levels may be also associated with the number and type of metastatic site, including in NSCLC (11, 26)​. In our adenocarcinoma subgroup, patients with n=3/4 metastatic sites had higher cEV miR-106b and miR-451a compared to those with n=1/2 metastatic sites and its expression level was also associated with metastatic spread in bones. In particular, miR-106b over-expression has been stated in multiple tumor types, such as breast, prostate and gastric, and it was demonstrated that it controls metastases in addition to cell proliferation, migration and invasion (29). Recently, it was reported that the expression of miR-106a stimulates bone metastasis of lung adenocarcinoma and its expression, by reducing PTEN expression, may be a new therapeutic target for bone metastasis in adenocarcinoma (63, 64). miR-106b directly regulates PTEN mRNA and protein expression in lung cancer cells increasing cell migration and invasion ability. In addition, this miRNA promotes proliferation and caspase-mediated cell death inhibition by negatively regulating BTG anti-proliferation factor 3 (BTG3) which suppress proliferation and metastasis (29).

As regards miR-451a, it was reported as a strongly down-regulated miRNA in NSCLC tumor tissues and its low expression seemed to be related to poor tumor differentiation, advanced pathological stage and lymph node metastasis (43). Thus, our findings could denote that miR-106b and miR-451a had an important role in metastasis formation, as an indicator not only of site number but also of organ type affected by metastases.

In addition, by restricting the analyses of PFS, OS and metastasis to adenocarcinoma subtype NSCLC, our data increased in significance by going in the direction of the miRNAs as potential classifier of specific NSCLC subtype. These findings are very encouraging to continue this path. Several studies identified both free circulating form and tumor tissue-derived miRNAs as classifiers of specific NSCLC subtype (adeno or squamous) (65, 66). This ability of miRNAs is due to the genetic and epigenetic composition of each lung cancer subtype, resulting in distinct cellular signaling pathways and phenotypic differences regulated by these molecules. We hypothesized that the NSCLC subtype-specific expression of miRNAs might also be reflected in serum EVs of patients. Therefore, we analyzed selected cEV-miRNAs in the two subtypes of our NSCLC case series (**Supplementary Figure S4B**). Our data revealed non-significant expression trends between miRNA expression and specific subtypes probably due to the low number of samples (n=16 adeno vs n=5 squamous).

Interestingly, in the adenocarcinoma subgroup, instead we have found the association between miR-10a, miR-22 and miR-34a with immunotherapy response and PFS prognosis. To our knowledge, few information about miR-10a and miR-22 are related only to NSCLC tissue and serum. In particular, a pilot miRNA profiling revealed that serum miR-10a was down-regulated in patients which respond to Nivolumab compared to non-responder ones (14). Besides, miR-10a is over-expressed in NSCLC tissue and serum, and it correlated with clinical stage and tumor metastasis (18, 19). Regarding miR-22, it was recently reported that its level dramatically decreased in lung cancer tissues and cells compared to their normal counterparts and its expression was also correlated with lymph node metastasis and tumor size, but not TNM stages (23). Thus, our data on miR-10a and miR-22 could open a new prospectives in NSCLC context.

Instead, miR-34a role as both predictive and prognostic biomarker was previously described, reporting that higher miR-34a levels were associated with better response to nivolumab, better OS (26) and better DFS (27). In these papers, miR-34 was quantified as free form in plasma of two NSCLC cohorts, one with advanced multi-treated patients and another with patients that received a complete tumor resection without any preoperative adjuvant therapy, respectively (26, 27). The dissimilar role of miR-34a, in immunotherapy response and in the survival analysis, reported in our study compared to above papers, could be due to both different miRNA-source (serum cEV vs plasma-free form) and patients’ characteristics (untreated vs treated/subjected to surgery). Therefore, we believe that miR-34a expression could be affected by previous treatments. This hypothesis was supported by other studies which demonstrated that plasma miR-34a levels changed after chemotherapy in breast cancer and osteosarcoma (67–69).

In addition to all miRNA data, in our NSCLC cohort, patients with ECOG PS=1 and=2 had significantly lower amount of EVs compared to those with ECOG PS=0. Our hypothesis is that the release of EVs is affected by advancing disability, passing from 0 to 1 or 2. Several recent studies have shown that cEV concentrations rapidly increased during physical exercise, thus raising a great interest about their roles in systemic adaptation to exercise and disease prevention (70, 71).

Serum-derived EV biomarkers could be integrated into diagnostic workflows, including companion diagnostics, using them as biomarkers to detect the presence of disease, predict patient outcomes, or monitor treatment response. In companion diagnostics, cEVs can be used to identify and follow patients over time who may benefit from a particular treatment or to predict treatment resistance. However, several carefully designed clinical-translational studies are needed to establish the diagnostic accuracy and clinical utility of EV-based liquid biopsy in oncology. The enumeration of EVs and/or their specific molecular characteristics should be included in clinical trials as diagnostic tests or for stage/subtype identification and as companion diagnostic tests i) to evaluate new therapeutic agents, ii) to guide therapeutic decision-making and iii) to monitor response to therapy.

Undoubtedly, EV-liquid biopsy has different advantages if compared with tissue/tumor biopsies. Firstly, tissue biopsies may not reflect the comprehensive situation of the disease due to tumor heterogeneity, while liquid biopsies can help to provide a general assessment. Secondly, with advances in clinical practice, EV-liquid biopsies may greatly reduce the number of invasive biopsies. Dynamic assessment of circulating EV-biomarkers could be used to guide long-term clinical decisions. In the specific case of EV-derived miRNAs, their quantization could be implemented in clinical practice by complementing the analysis of tumor biopsy based on PD-L1 to lead therapeutic decisions.

Overall, our study had several limitations. First, it’s a single-center study with a small NSCLC sample size which may limit the generalizability of the results. Thus, a larger, multicenter patient validation cohort would provide more robust results and increase the statistical power of analysis. Second, the confounding factors (e.g., smoking history, KRAS/EGFR status) were not fully adjusted due to the small sample size. In addition, our study highlighted that some miRNAs are involved in pembrolizumab response but did not identify the mechanisms by which they influence therapy response. Functional studies are needed to confirm the biological relationship between miRNAs and NSCLC pathways. Furthermore, this study may not have captured the full spectrum of responses to other immunotherapy drugs or combined therapies. Nevertheless, many data regarding the relationship between miRNA expression and clinical features were statistically significant and it was very encouraging. Finally, for clinical translationality, it will be crucial to confirm our findings in a large validation cohort.

**Figure 7:**
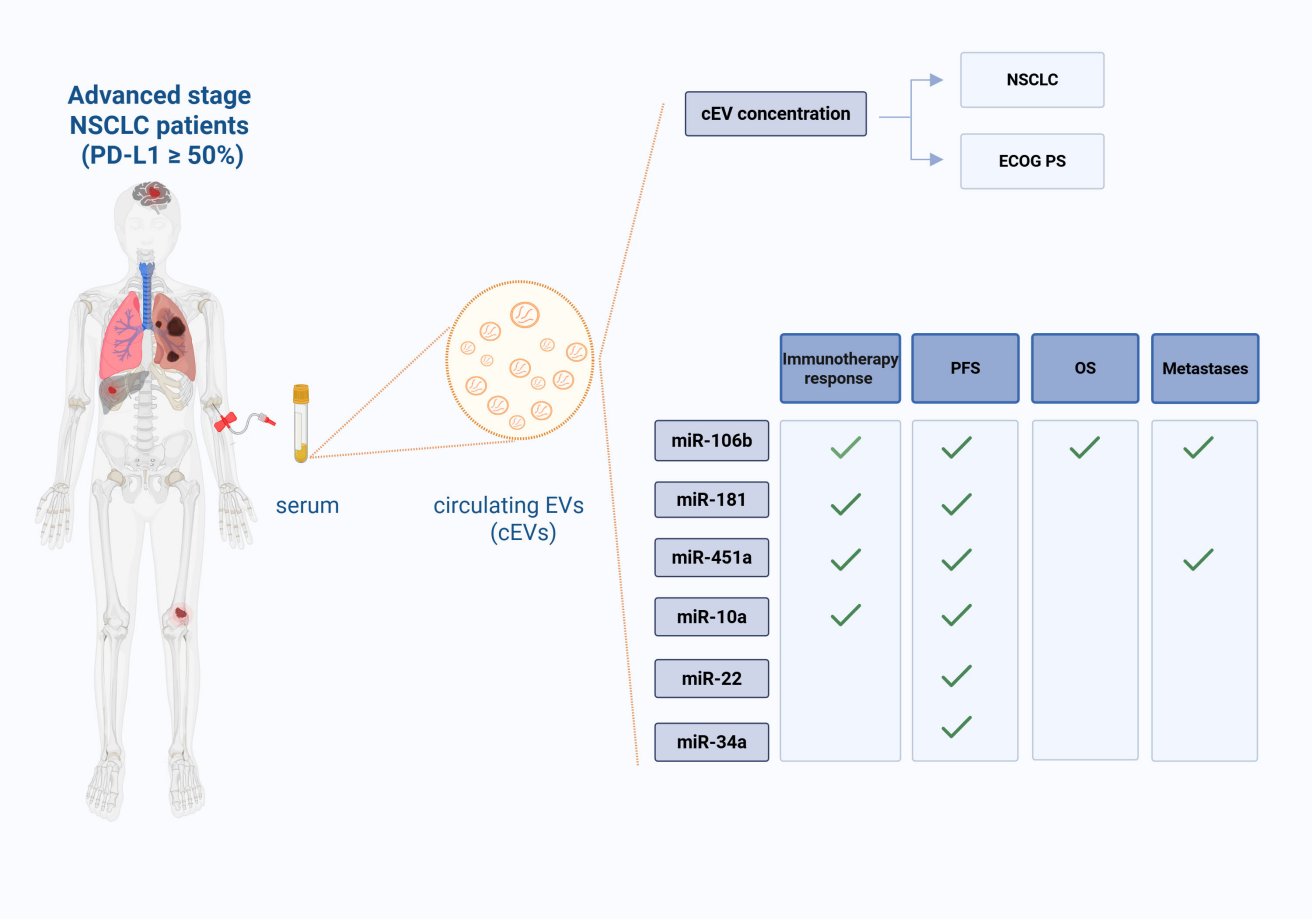
Liquid biopsy in advanced stage NSCLC patients (with PD-L1 expression ≥50%): role of serum cEVs and of their associated miRNAs. Circulating EVs (cEVs) were isolated from serum of metastatic advanced stage NSCLC patients at diagnosis, before first-line immunotherapy treatment. cEV concentration was associated to NSCLC diagnosis and ECOG PS scale; cEV miRNA levels were associated to treatment response, Progression Free Survival (PFS), Overall Survival (OS) and number of metastases. This figure was created with BioRender.com.

In conclusion, the discovery of highly sensitive and accurate non-invasive biomarkers by EV liquid biopsy could be a promising approach in NSCLC. More interesting, there are already different clinical trials employing liquid biopsy and miRNAs in lung cancer (NCT04529915, NCT04182893, NCT03108677; clinicaltrials.gov). In particular, an ongoing clinical trial (ClinicalTrials.gov identifier: NCT04427475) aimed to predict immunotherapy response using PD-L1 exosome and their miRNAs in NSCLC. In this context, our study could provide the first proof-of-concept that serum cEV miRNAs, in a cohort of advanced stage NSCLC patients with high PD-L1 expression, could be new biomarkers for NSCLC diagnosis, for pembrolizumab response prediction, for both PFS and OS prognosis and for metastases investigation (**Figure 7**). Independent validations on larger cohorts are needed to confirm our findings.

## Data Availability

The raw data supporting the conclusions of this article will be made available by the authors, without undue reservation.
