# Supplementary material for: Serum extracellular vesicle microRNAs as potential biomarkers to predict pembrolizumab response and prognosis in metastatic non-small cell lung cancer patients
Source: Front Immunol. 2025 Jun 4;16:1540906. doi: 10.3389/fimmu.2025.1540906 (PMC12174437; doi:10.3389/fimmu.2025.1540906)
Supplement: Supplementary file 1 [file DataSheet1.zip › Supplementary Table S1 and S3.DOCX]

**Supplementary Table 1:** Median expression of serum EV miRNAs in advanced stage adenocarcinoma NSCLC patients.

| **miRNAs** | **Median Expression (copies/µl)**  n= 21 NSCLC | **Median Expression (copies/µl)**  n= 16 adenocarcinoma NSCLC |
| --- | --- | --- |
| miR-10a-5p | 1080 | 1190 |
| miR-21-5p | 112000 | 102000 |
| miR-22-5p | 1260 | 1330 |
| miR-30a-5p | 37000 | 36500 |
| miR-34a-5p | 300 | 350 |
| miR-106b-5p | 10000 | 10600 |
| miR-125b-5p | 7000 | 6700 |
| miR-150-5p | 21200 | 21150 |
| miR-155-5p | 460 | 470 |
| miR-181a-5p | 12200 | 11150 |
| miR-181b-5p | 460 | 430 |
| miR-451a | 1480000 | 1590000 |

**Supplementary Table 3:** miRNA combinations and relative targets.

| n° of miRNA | miRNAs | Target |
| --- | --- | --- |
| 6 | hsa-mir-10a-5p, hsa-mir-21-5p, hsa-mir-106b-5p, hsa-mir-155-5p, hsa-mir-181a-5p, hsa-mir-181b-5p | PTEN |
| 6 | hsa-mir-21-5p, hsa-mir-34a-5p, hsa-mir-125b-5p, hsa-mir-181a-5p, hsa-mir-181b-5p, hsa-mir-451a | BCL2 |
| 4 | hsa-mir-34a-5p, hsa-mir-155-5p, hsa-mir-181a-5p, hsa-mir-181b-5p | FOS |
| 4 | hsa-mir-10a-5p, hsa-mir-106b-5p, hsa-mir-181a-5p, hsa-mir-181b-5p | BCL2L11 |
| 4 | hsa-mir-21-5p, hsa-mir-34a-5p, hsa-mir-106b-5p, hsa-mir-181b-5p | E2F1 |
| 4 | hsa-mir-30a-5p, hsa-mir-34a-5p, hsa-mir-125b-5p, hsa-mir-150-5p | TP53 |
| 4 | hsa-mir-21-5p, hsa-mir-106b-5p, hsa-mir-125b-5p, hsa-mir-181a-5p | STAT3 |
| 3 | hsa-mir-34a-5p, hsa-mir-155-5p, hsa-mir-451a | MYC |
| 3 | hsa-mir-106b-5p, hsa-mir-125b-5p, hsa-mir-451a | MMP2 |
| 3 | hsa-mir-106b-5p, hsa-mir-125b-5p, hsa-mir-155-5p | APC |
| 3 | hsa-mir-125b-5p, hsa-mir-181a-5p, hsa-mir-181b-5p | MCL1 |
| 3 | hsa-mir-21-5p, hsa-mir-106b-5p, hsa-mir-155-5p | VEGFA |
| 3 | hsa-mir-21-5p, hsa-mir-125b-5p, hsa-mir-181b-5p | IGF1R |
| 3 | hsa-mir-10a-5p, hsa-mir-21-5p, hsa-mir-155-5p | BCL6 |
| 3 | hsa-mir-34a-5p, hsa-mir-106b-5p, hsa-mir-155-5p | CCND1 |
| 3 | hsa-mir-34a-5p, hsa-mir-125b-5p, hsa-mir-451a | AKT1 |
| 3 | hsa-mir-106b-5p, hsa-mir-181a-5p, hsa-mir-181b-5p | KAT2B |
| 3 | hsa-mir-21-5p, hsa-mir-34a-5p, hsa-mir-181a-5p | HMGB1 |
| 3 | hsa-mir-30a-5p, hsa-mir-34a-5p, hsa-mir-181a-5p | NOTCH1 |
| 3 | hsa-mir-21-5p, hsa-mir-34a-5p, hsa-mir-125b-5p | ERBB2 |
| 3 | hsa-mir-34a-5p, hsa-mir-181a-5p, hsa-mir-181b-5p | SIRT1 |
| 3 | hsa-mir-34a-5p, hsa-mir-181a-5p, hsa-mir-451a | IL6R |
| 3 | hsa-mir-34a-5p, hsa-mir-150-5p, hsa-mir-155-5p | MYB |
| 3 | hsa-mir-34a-5p, hsa-mir-181a-5p, hsa-mir-181b-5p | MAP2K1 |
