# Supplementary material for: Serum extracellular vesicle microRNAs as potential biomarkers to predict pembrolizumab response and prognosis in metastatic non-small cell lung cancer patients
Source: Front Immunol. 2025 Jun 4;16:1540906. doi: 10.3389/fimmu.2025.1540906 (PMC12174437; doi:10.3389/fimmu.2025.1540906)
Supplement: Supplementary file 1 [file DataSheet1.zip › Supplementary Figures.PPTX]

## Slide 1
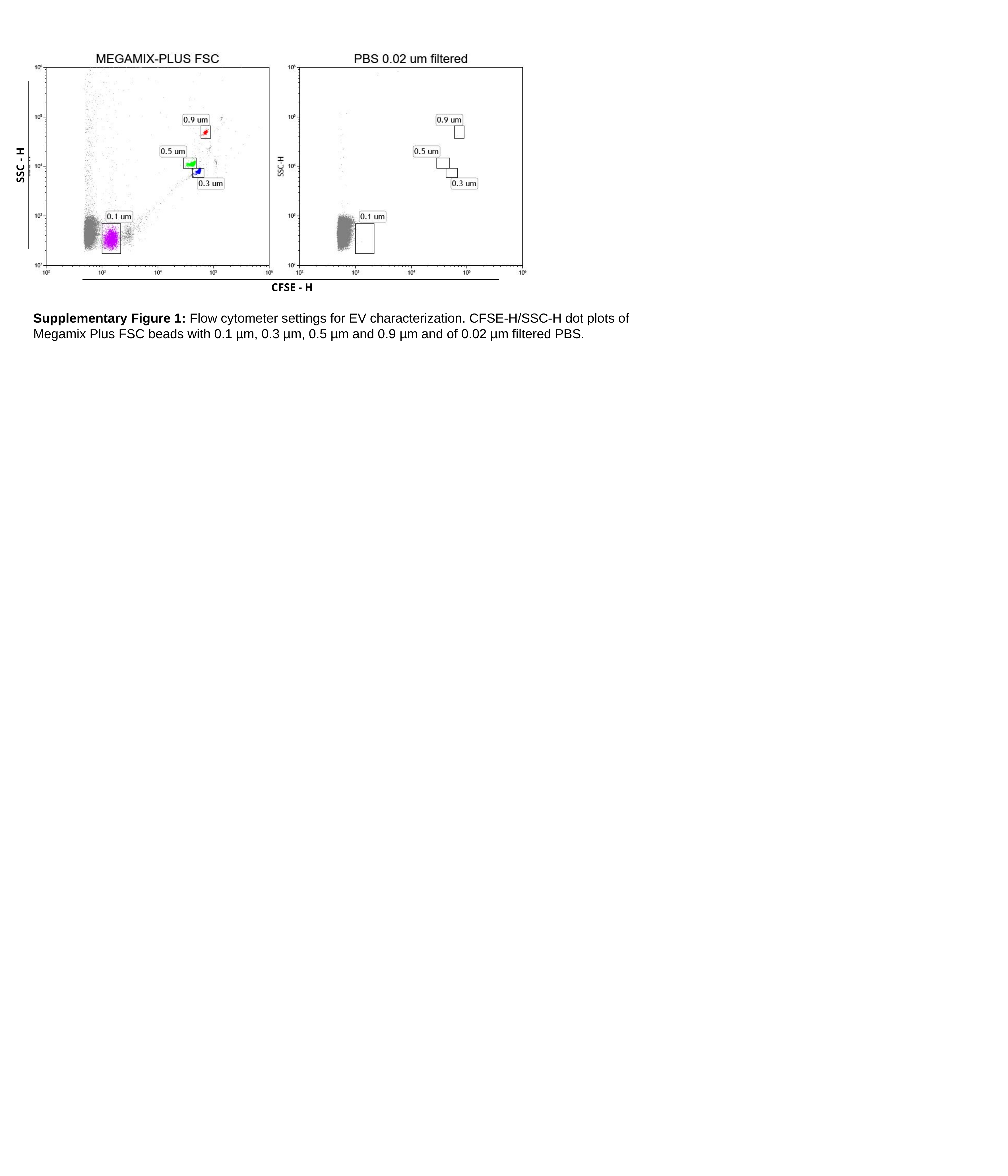

SSC - H
CFSE - H
Supplementary Figure 1: Flow cytometer settings for EV characterization. CFSE-H/SSC-H dot plots of Megamix Plus FSC beads with 0.1 µm, 0.3 µm, 0.5 µm and 0.9 µm and of 0.02 µm filtered PBS.

## Slide 2
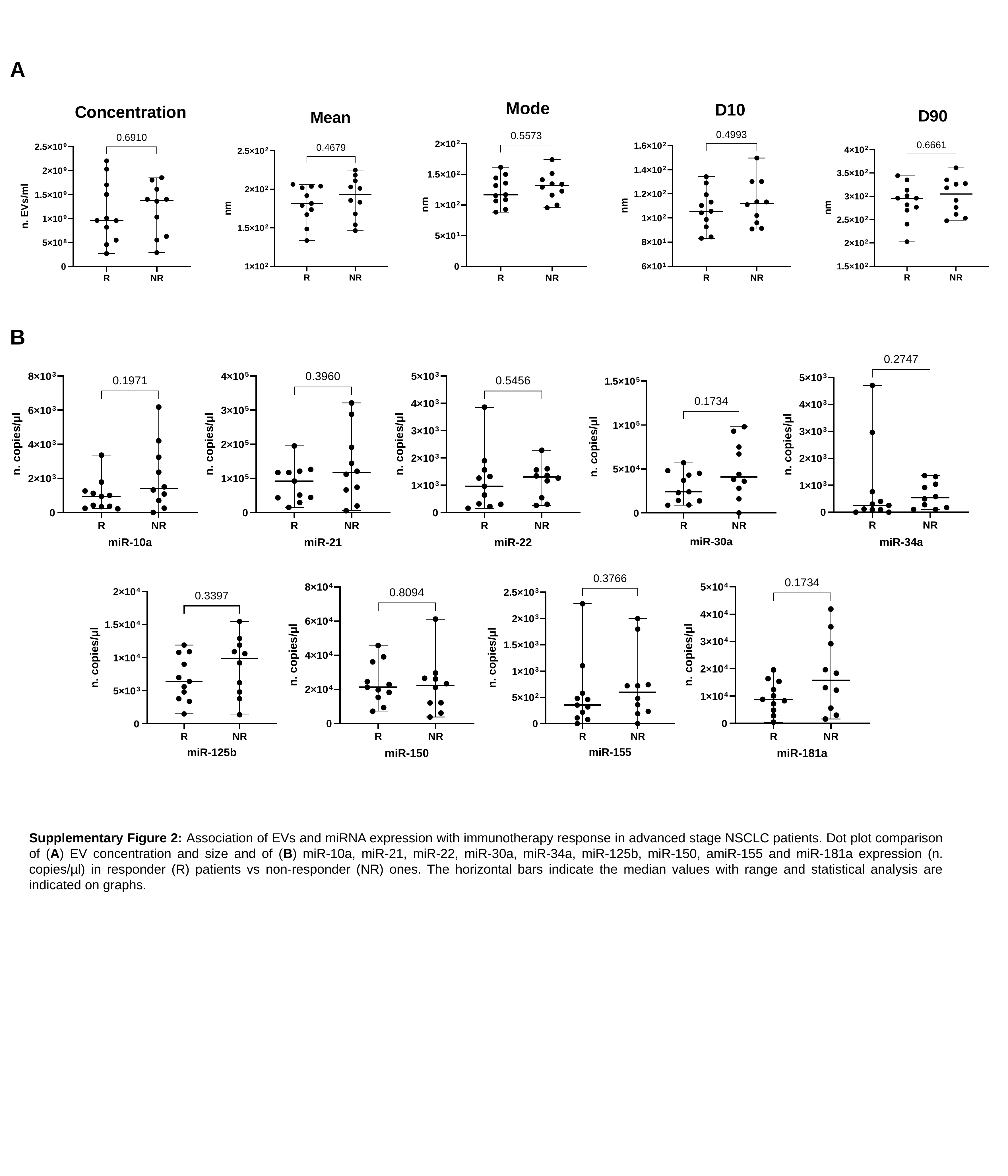

A
B
Supplementary Figure 2: Association of EVs and miRNA expression with immunotherapy response in advanced stage NSCLC patients. Dot plot comparison of (A) EV concentration and size and of (B) miR-10a, miR-21, miR-22, miR-30a, miR-34a, miR-125b, miR-150, amiR-155 and miR-181a expression (n. copies/µl) in responder (R) patients vs non-responder (NR) ones. The horizontal bars indicate the median values with range and statistical analysis are indicated on graphs.

## Slide 3
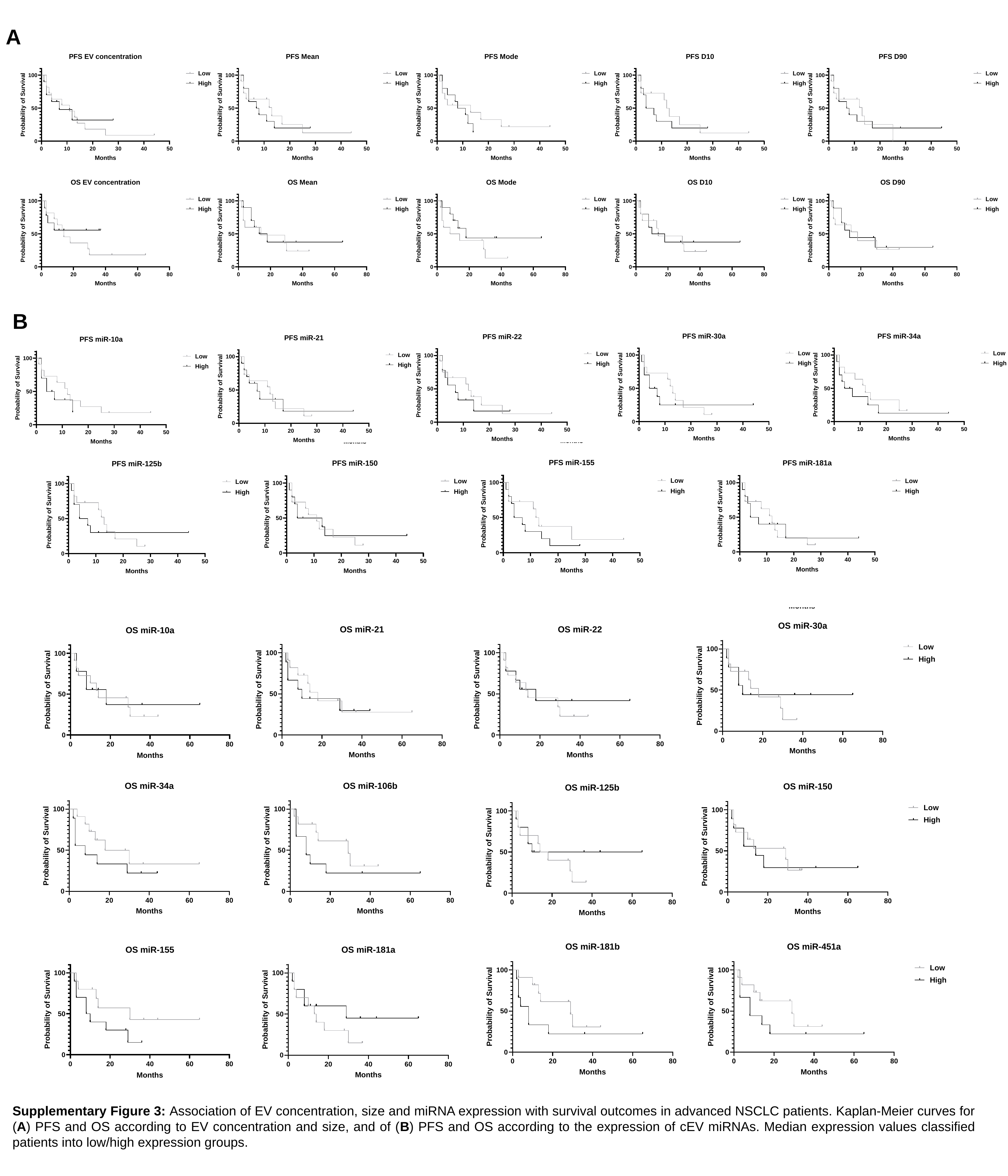

A
B
Supplementary Figure 3: Association of EV concentration, size and miRNA expression with survival outcomes in advanced NSCLC patients. Kaplan-Meier curves for (A) PFS and OS according to EV concentration and size, and of (B) PFS and OS according to the expression of cEV miRNAs. Median expression values classified patients into low/high expression groups.

## Slide 4
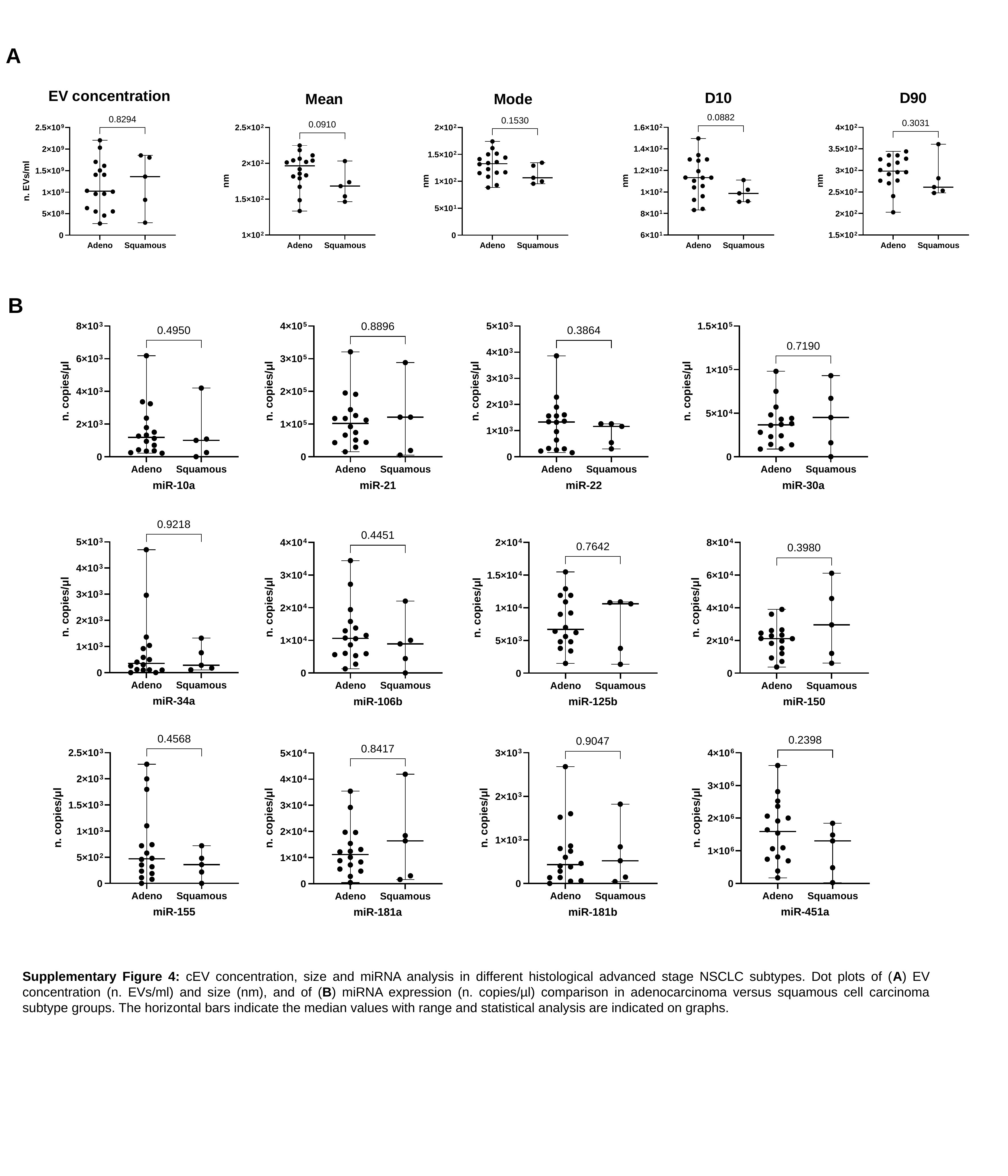

A
B
Supplementary Figure 4: cEV concentration, size and miRNA analysis in different histological advanced stage NSCLC subtypes. Dot plots of (A) EV concentration (n. EVs/ml) and size (nm), and of (B) miRNA expression (n. copies/µl) comparison in adenocarcinoma versus squamous cell carcinoma subtype groups. The horizontal bars indicate the median values with range and statistical analysis are indicated on graphs.

## Slide 5
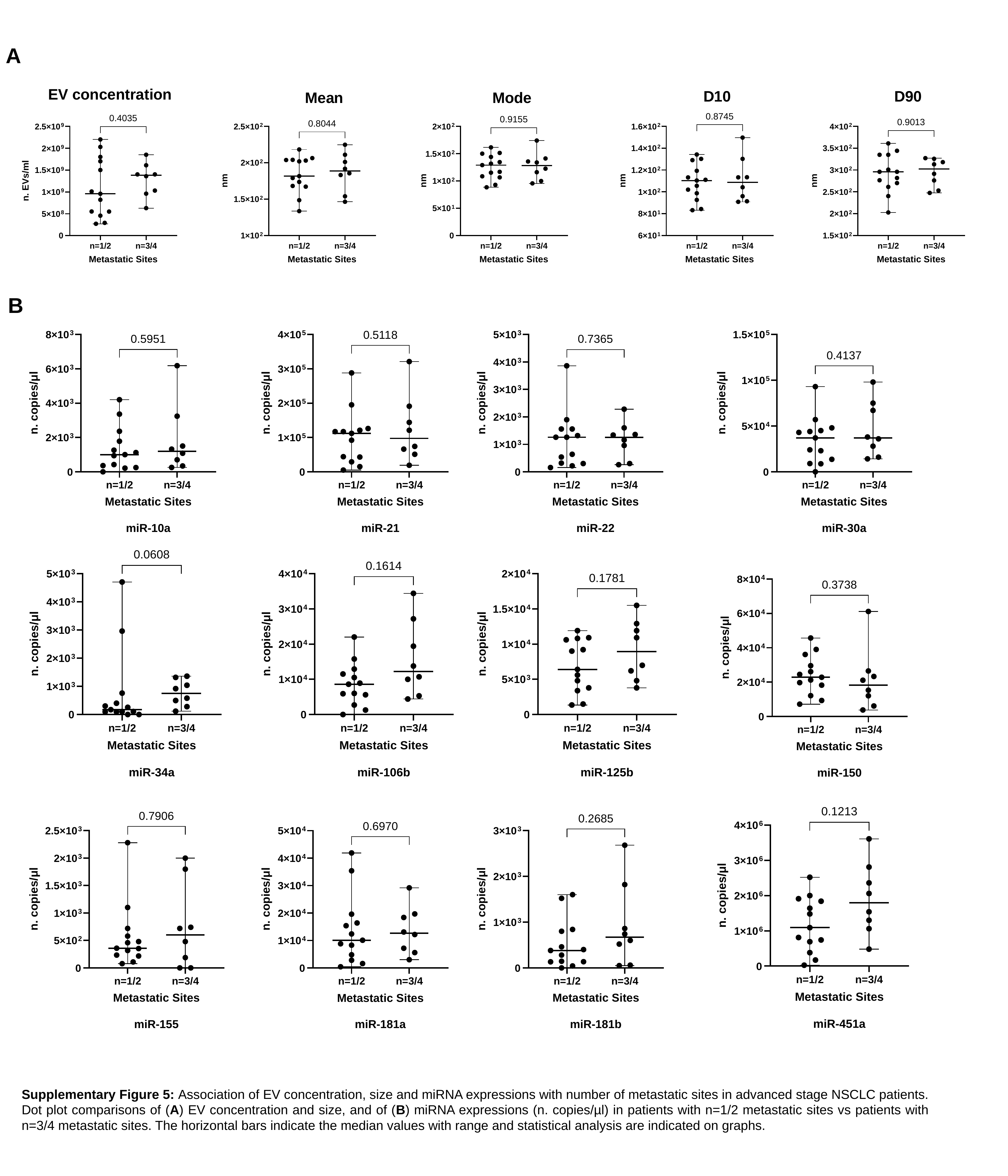

A
B
Supplementary Figure 5: Association of EV concentration, size and miRNA expressions with number of metastatic sites in advanced stage NSCLC patients. Dot plot comparisons of (A) EV concentration and size, and of (B) miRNA expressions (n. copies/µl) in patients with n=1/2 metastatic sites vs patients with n=3/4 metastatic sites. The horizontal bars indicate the median values with range and statistical analysis are indicated on graphs.

## Slide 6
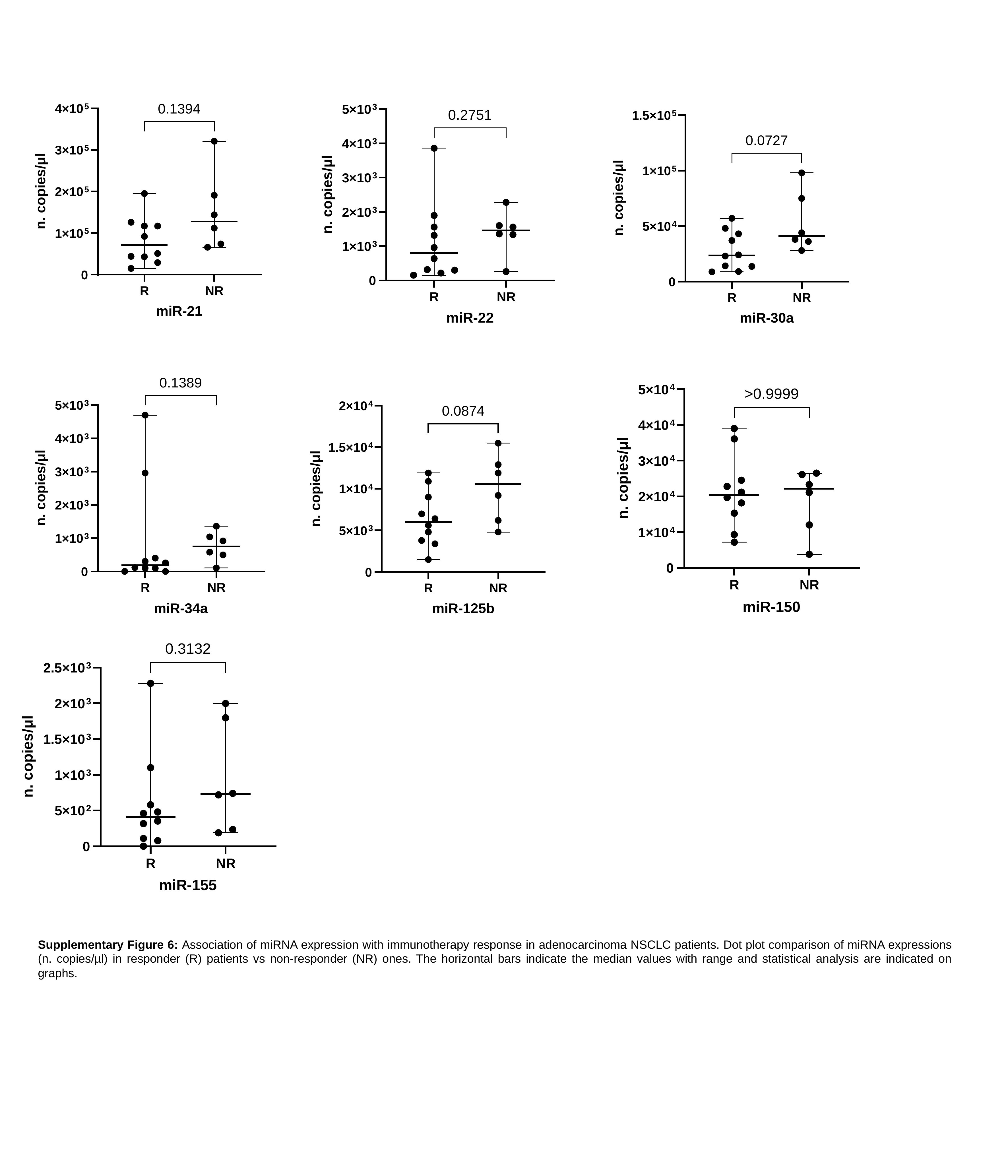

Supplementary Figure 6: Association of miRNA expression with immunotherapy response in adenocarcinoma NSCLC patients. Dot plot comparison of miRNA expressions (n. copies/µl) in responder (R) patients vs non-responder (NR) ones. The horizontal bars indicate the median values with range and statistical analysis are indicated on graphs.

## Slide 7
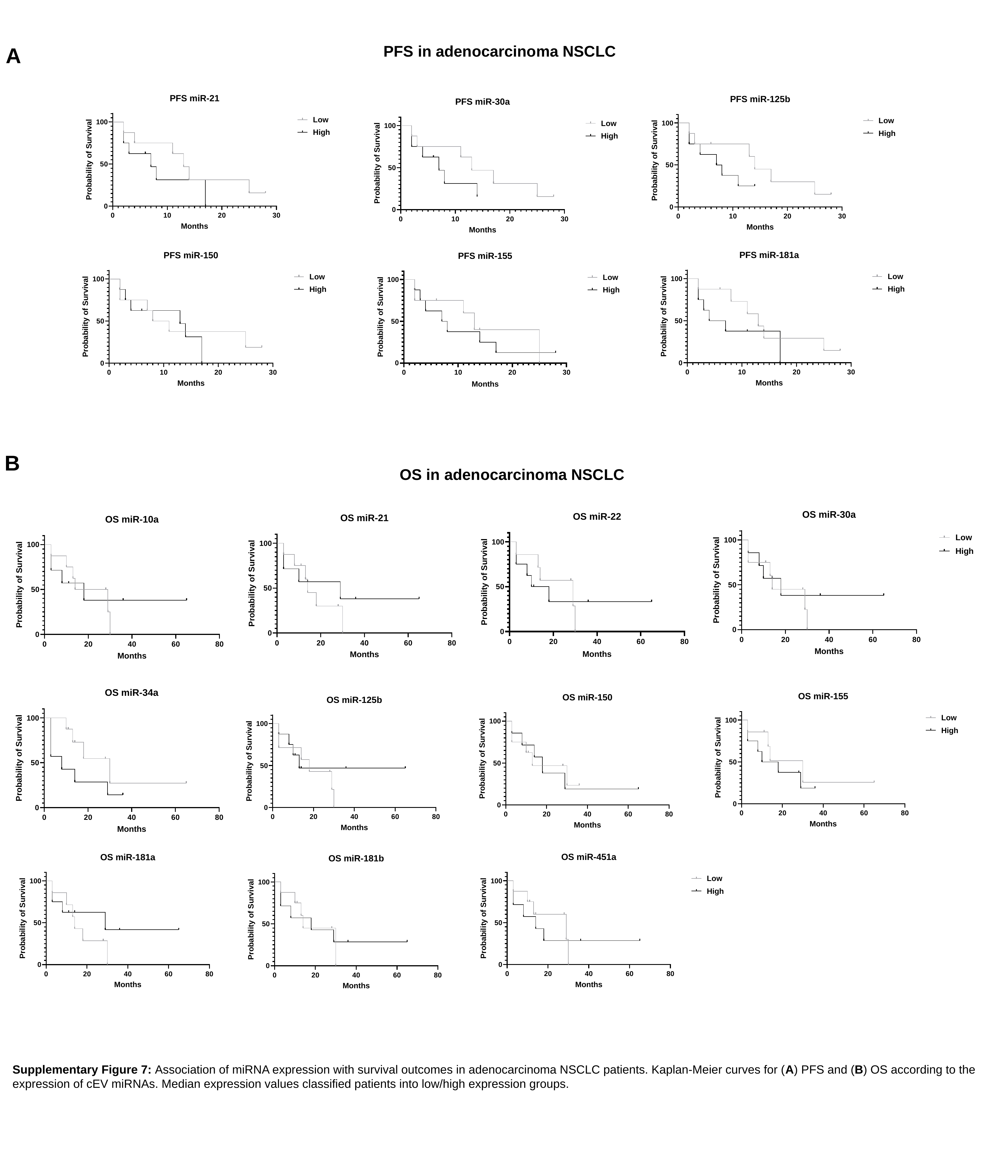

PFS in adenocarcinoma NSCLC
A
B
OS in adenocarcinoma NSCLC
Supplementary Figure 7: Association of miRNA expression with survival outcomes in adenocarcinoma NSCLC patients. Kaplan-Meier curves for (A) PFS and (B) OS according to the expression of cEV miRNAs. Median expression values classified patients into low/high expression groups.

## Slide 8
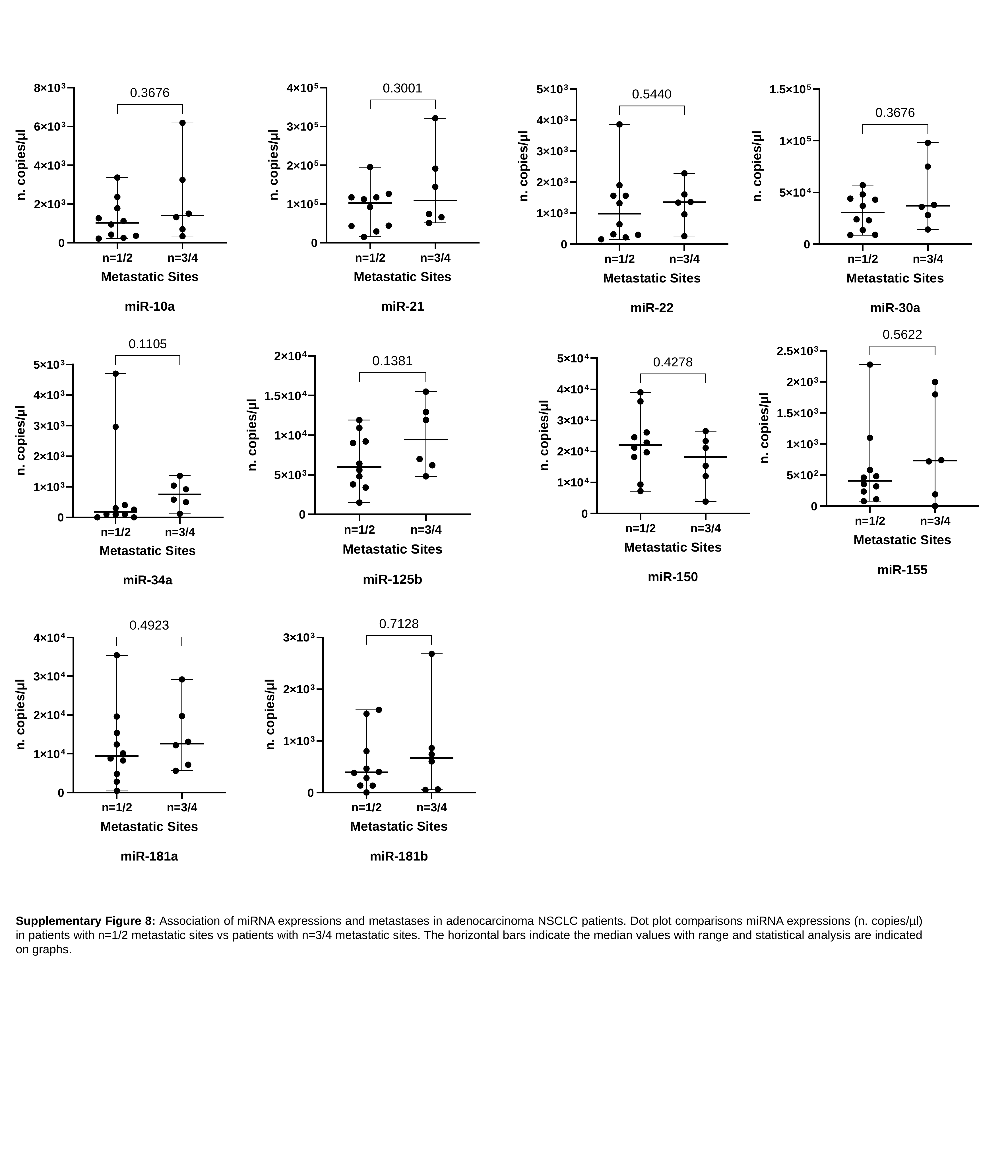

Supplementary Figure 8: Association of miRNA expressions and metastases in adenocarcinoma NSCLC patients. Dot plot comparisons miRNA expressions (n. copies/µl) in patients with n=1/2 metastatic sites vs patients with n=3/4 metastatic sites. The horizontal bars indicate the median values with range and statistical analysis are indicated on graphs.
